# Thermodynamic insights into 2-thiouridine-enhanced RNA hybridization

**DOI:** 10.1093/nar/gkv761

**Published:** 2015-08-03

**Authors:** Aaron T. Larsen, Albert C. Fahrenbach, Jia Sheng, Julia Pian, Jack W. Szostak

**Affiliations:** 1 Howard Hughes Medical Institute, Center for Computational and Integrative Biology, and Department of Molecular Biology, Simches Research Center, Massachusetts General Hospital, Boston, MA 02114, USA; 2 Earth-Life Science Institute, Tokyo Institute of Technology, 2-12-1-IE-1 Ookayama, Meguro-ku, Tokyo 152-8550, Japan; 3 University at Albany, State University of New York, Department of Chemistry, The RNA Institute, 1400 Washington Avenue, Albany, NY 12222, USA

## Abstract

Nucleobase modifications dramatically alter nucleic acid structure and thermodynamics. 2-thiouridine (s^2^U) is a modified nucleobase found in tRNAs and known to stabilize U:A base pairs and destabilize U:G wobble pairs. The recently reported crystal structures of s^2^U-containing RNA duplexes do not entirely explain the mechanisms responsible for the stabilizing effect of s^2^U or whether this effect is entropic or enthalpic in origin. We present here thermodynamic evaluations of duplex formation using ITC and UV thermal denaturation with RNA duplexes containing internal s^2^U:A and s^2^U:U pairs and their native counterparts. These results indicate that s^2^U stabilizes both duplexes. The stabilizing effect is entropic in origin and likely results from the s^2^U-induced preorganization of the single-stranded RNA prior to hybridization. The same preorganizing effect is likely responsible for structurally resolving the s^2^U:U pair-containing duplex into a single conformation with a well-defined H-bond geometry. We also evaluate the effect of s^2^U on single strand conformation using UV- and CD-monitored thermal denaturation and on nucleoside conformation using ^1^H NMR spectroscopy, MD and umbrella sampling. These results provide insights into the effects that nucleobase modification has on RNA structure and thermodynamics and inform efforts toward improving both ribozyme-catalyzed and nonenzymatic RNA copying.

## INTRODUCTION

RNA plays an essential and diverse role in living systems, acting as genetic information carrier, catalyst and regulator (1–4). Functional RNAs adopt many well-defined 3D structures resulting from specific base–base interactions including normal Watson–Crick base pairs and a variety of other associations (5). Understanding the structure and thermodynamics of base–base interactions provides a foundation for elucidating RNA structure/function relationships, engineering novel applications such as RNA-based therapeutics and addressing questions related to the origins of life (6).

Nucleobase modification diversifies nucleic acid structure and function (7). The significance of this role is reflected by the fact that certain modified nucleobases are among the most highly conserved features of RNA and, for this reason, are regarded as chemical fossils of molecular evolution. Of the approximately 140 known post-transcriptional RNA modifications, 60 are specific to uridine (U) and 16 feature thiolation at the C2 position of U (8). These modified nucleobases include s^2^U and various 5-modified derivatives of s^2^U. While it is possible that some of these modifications are relics of the RNA world (9), the extent to which 2-thiolation is conserved at the anticodon site of tRNA suggests that this modification is of critical functional importance in translation (10). It has been demonstrated that s^2^U and its 5-modified derivatives enhance the efficiency and accuracy of codon-anticodon recognition, prevent frame-shifting during translation and improve tRNA aminoacylation kinetics (11). Improved codon-anticodon recognition resulting from 2-thiolation in the human tRNA^Lys3^_UUU_ has also proved to be important for reverse transcription of the HIV-1 viral genome (12).

Modified nucleobases (such as s^2^U) also address complications related to the RNA world hypothesis including protein-free nucleic acid copying (13). Despite the fact that *in vitro* selection has afforded an RNA-dependent RNA polymerase capable of producing a polymer longer than itself, this feat has been performed only on purposefully designed and highly optimized templates (14). Poor rate and fidelity currently prevent the complete copying of non-optimized or highly structured RNA templates, including the RNA polymerase ribozyme itself (6). Similar challenges also prevent the realization of nonenzymatic, template-directed nucleic acid replication systems capable of copying long templates. In these cases, self replication is limited in part by the frequent incorporation of mismatched base-pairs, occurring at rates as high as once for every 5–6 nucleotides incorporated in some systems (15). In addition to compromising the fidelity of the resulting genetic message, some mismatches also strongly inhibit subsequent polymerization, potentially halting further extension (16).

Issues of rate and fidelity can be addressed by the use of modified nucleobases such as s^2^U. 2-Thiolation has been demonstrated to stabilize Watson–Crick U:A base pairs and destabilize U:G wobble-pairs, the most commonly observed mismatch in nonenzymatic RNA copying (17–19). We recently exploited these effects to enhance the rate and fidelity of nonenzymatic, template-directed RNA copying (20).

**Figure 1.**
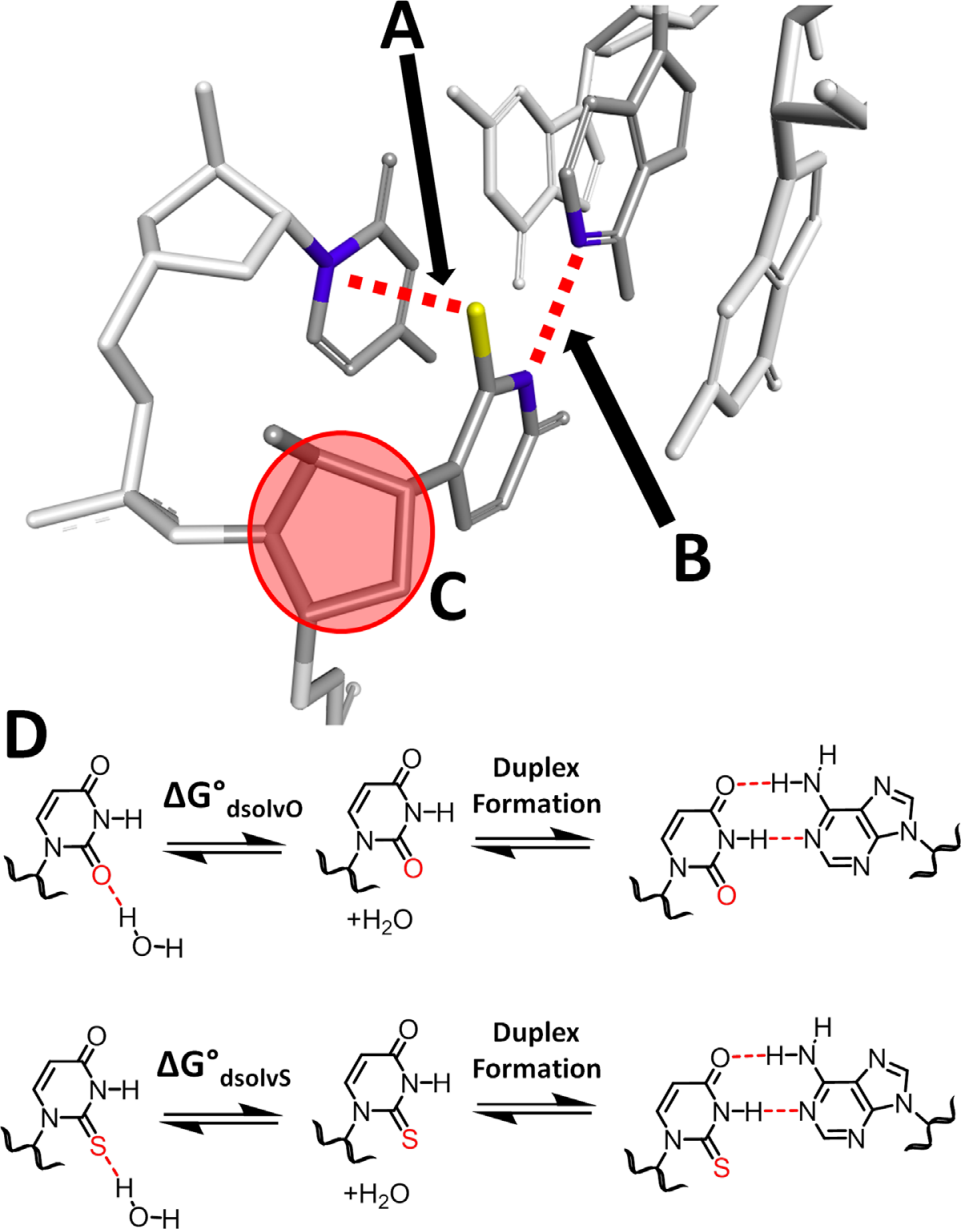
The proposed mechanisms of s^2^U-induced stabilization of duplex RNA: S may (**A**) enhance S/N1 stacking due to the more polarizable nature of S; (**B**) increase N3 H-bonding due to the increased acidity of the N3 imino proton; (**C**) stabilize the 3′-*endo* sugar pucker (circled in red); the structure was adapted from PDB ID: 4U34 (27); and/or (**D**) reduce the desolvation penalty incurred during duplex formation.

Although several thermodynamic (21) and NMR (17) studies have evaluated the effects of s^2^U on base-pairing in RNAs, the actual mechanism by which s^2^U affects RNA structure and thermodynamics remains unclear. Current mechanistic proposals include (i) enhanced base stacking, particularly S/N1 stacking due to the highly polarizable nature of sulfur (22); (ii) stronger N3 H-bonding as a result of the increased acidity of the N3 imino proton (17,23); (iii) sulfur-induced stabilization of the 3′-*endo* sugar pucker leading to enhanced conformational ordering (24); and (iv) a reduced desolvation penalty incurred during duplex formation owing to the relatively poor H-bonding ability of sulfur (25) (Figure 1). It is conceivable that any and all of these mechanisms may contribute to s^2^U-induced duplex stabilization to differing degrees, and in particular, mechanisms (i) and (iii) are both likely to enhance preorganization (26) of the single strand prior to duplex formation.

**Figure 2.**
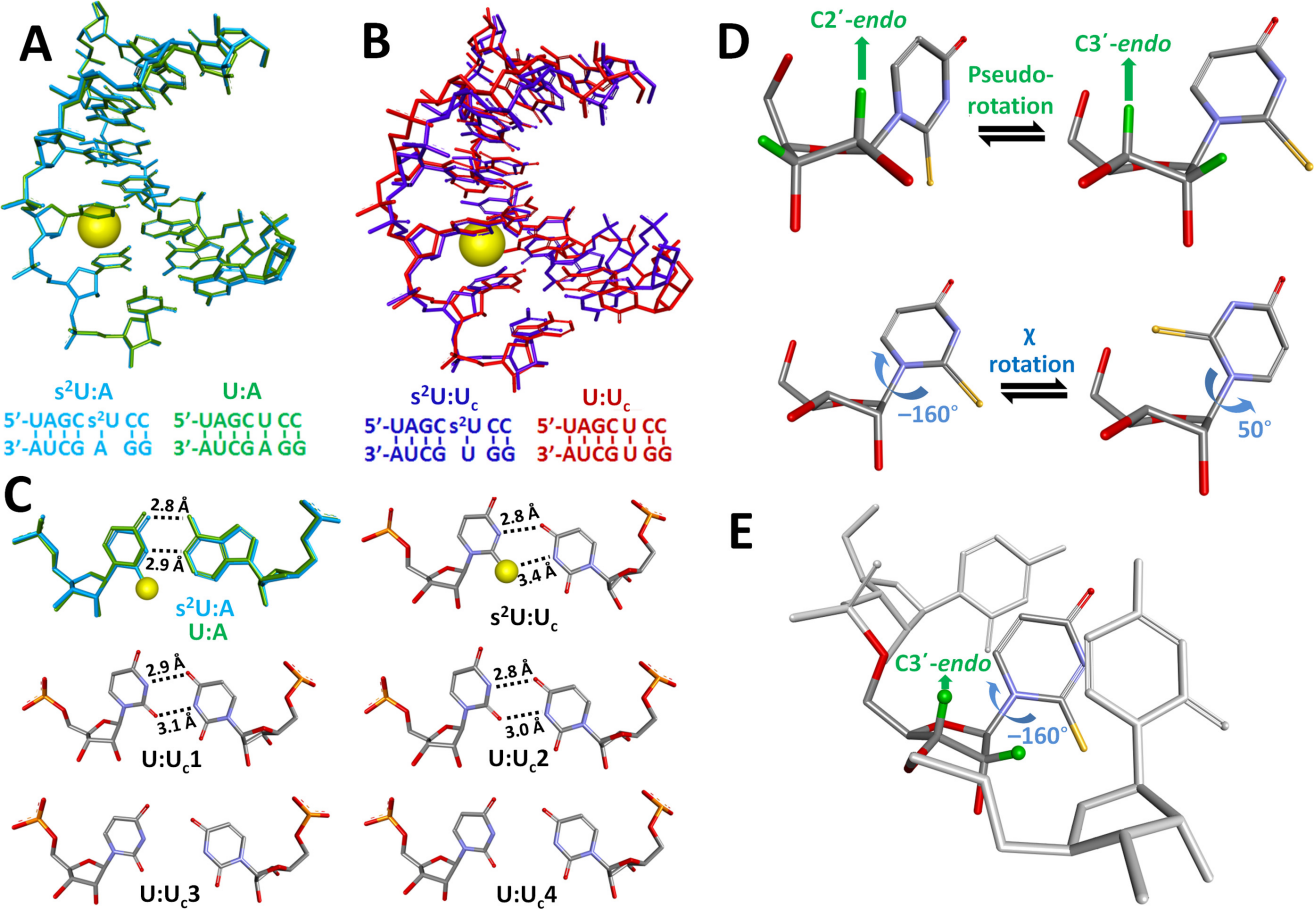
Crystal structures, sequences and structural features of the RNA duplexes investigated here; (**A**) Structural comparison of **s^2^U:A** (red, PDB ID: 4U34) and **U:A** (blue, PDB ID: 4U37), RMSD is < 0.2 Å; (**B**) Structural comparison of **s^2^U:U_c_** (light blue, PDB ID: 4U35) and **U:U_c_1** (pink, PDB ID: 4U38), the most structurally similar duplex from **U:U_c_**, RMSD is 1.3 Å. Sulfur atoms are displayed as yellow spheres scaled to their van der Waals radii; (**C**) A comparison of H-bond geometries of base pairs at the site of s^2^U-substitution. The geometries for **s^2^U:A** and **U:A** overlap well and the geometry for **s^2^U:U_c_** is most similar to that of **U:U_c_1**; (**D**) Pseudorotation phase angle (*P*) and chi torsion angle (χ) rotation of a representative s^2^U nucleotide; (**E**) s^2^U nucleotide in PDB ID: 4U34 adopting the C3′-*endo* sugar pucker and a χ value of –160°. These values are normally found in structured RNA as they maximize base–base stacking and minimize strain and clashes.

Despite a statement from Davis and Kumar in 1997 regarding the necessity of addressing these questions by examining high-resolution structures of s^2^U-containing double stranded RNA (dsRNA) (17), such structures have been reported only very recently (27). The overall structures of the dsRNA containing an internal s^2^U:A pair (**s^2^U:A**) and its native counterpart (**U:A**) differ by a root-mean-squared deviation (RMSD) of < 0.2 Å (Figure 2A). In contrast, the structure of the dsRNA containing an internal s^2^U:U pair (**s^2^U:U_c_**) differs significantly compared to its native counterpart (**U:U_c_**) (Figure 2B). The unit cell of the **s^2^U:U_c_** crystal features only a single duplex with standard Watson–Crick H-bond geometry at all other base pairs (Figure 2C). The unit cell of the **U:U_c_** crystal, however, features four different duplex structures (denoted by **U:U_c_1** through **U:U_c_4**) featuring distinct H-bond geometries at the U:U wobble pair, two of which display irregular H-bond lengths and angles that correspond to relatively weak H-bond strengths. These observations suggest that **U:U_c_** exists in several conformations of comparable energy, a feature which may be related to the low observed duplex stability resulting from the internal U:U mismatch.

Each of the above proposed mechanistic explanations for s^2^U-enhanced duplex stabilization can be expected to have different effects on the thermodynamic parameters of duplex formation, namely Δ*G*, Δ*H* and Δ*S*. Here, we present the thermodynamic parameters of duplex formation measured from isothermal titration calorimetry (ITC) and thermal denaturation experiments of two s^2^U-containing heptamer RNA duplexes: **s^2^U:A** and **s^2^U:U_c_** (5′-uagc**s^2^U**cc-3′ paired with 3′-aucg**A**gg-5′ and 3′-aucg**U**gg-5′, respectively) and their corresponding native structures, **U:A**, and **U:U_c_**. We also evaluate the conformational stability of the single-stranded RNA (ssRNA) containing s^2^U in comparison to its native counterpart using thermal denaturation monitored by UV absorbance and circular dichroism (CD) spectroscopies. Next, we evaluate the conformational effect of 2-thiolation on the sugar pucker of the free U nucleoside using ^1^H NMR spectroscopy, MD simulations and umbrella sampling. Finally, we determine and compare the desolvation penalties for s^2^U and U nucleosides. Our findings provide insights into the mechanisms responsible for the effect of s^2^U on duplex formation and guide future studies focused on the structural and energetic effects of nucleobase modifications, particularly in the context of nonenzymatic RNA copying.

## MATERIALS AND METHODS

### Materials

All RNA oligonucleotides were chemically synthesized at 1.0-μmol scales by solid-phase synthesis. The 2′-TBDMS-protected RNA phosphoramidites were obtained from ChemGenes and were dissolved in acetonitrile to a concentration of 0.1 M. Coupling was performed using 5-(benzylmercapto)-1H-tetrazole (5-BMT) (0.25 M) in acetonitrile over 10 min. 0.02 M I_2_ in THF/Py/H_2_O solution was used as oxidizing reagent to maintain the oxidation state of the 2-thio-modification during synthesis. All other reagents were obtained from Glen Research. Synthesis was performed on the appropriate nucleoside immobilized via a succinate linker to controlled pore glass (CPG-500). All oligonucleotides were prepared in DMTr-off form. After synthesis, RNAs were cleaved from the solid support and fully deprotected with NH_4_OH:EtOH (3:1 v/v) at 55°C overnight. The solvent was completely removed by Speed-Vac concentration and the dried material was treated with 1 ml of Et_3_N•3HF at room temperature for 8 h. The reaction was quenched with 1 ml of water and the RNA was precipitated by adding 0.2 ml of 3 M sodium acetate and 6 ml of *n*-butanol. The solution was cooled to –30°C for 1 h before the RNA was recovered by centrifugation and finally dried *in vacuo* before purification by reverse-phase HPLC (Agilent 1100 series LC) on a Zorbax ExtendC18 column 9.4 mm × 250 mm, 5 μ particle size (Agilent Technologies) equilibrated with 30 mM triethylammonium bicarbonate/2% acetonitrile, pH 8.0, and eluted with an acetonitrile gradient. Purified nucleotides were lyophilized to dryness and stored at −80°C.

### Isothermal titration calorimetry (ITC)

All ITC experiments were performed using a TA Instruments Nano ITC. Samples were prepared by diluting stock solutions of HPLC-purified oligonucleotides into buffers containing 200 mM NaHEPES, pH 7.5 and 100 mM of NaCl. Oligonucleotide concentrations were verified by UV absorbance at 260 nm using extinction coefficients predicted by primary sequence. 50 μl samples of oligonucleotide solutions at various concentrations were titrated into the sample cell containing 170 μl of the complementary oligonucleotide at concentrations typically 5–10 fold lower. Samples were stirred at 350 rpm and 25°C with 220 s between injections of 1.5–2.5 μl. The values of Δ*H*, *K*_d_ and stoichiometry (*n*) were measured for the **s^2^U:A** and **U:A** duplexes by fitting the data to a model described previously (28). Because of significant self-dimerization observed for 3′-aucg**U**gg-5′ by optical melting, a more sophisticated model was required to afford thermodynamic parameters for **s^2^U:U_c_** and **U:U_c_** duplexes. Details of this model can be found in the SI. In all cases, Δ*S* was calculated using the relationship: Δ*G* = *RT* ln(*K*_d_) = Δ*H* –*T*Δ*S* (where *R* is the gas constant and *T* is temperature). For ease of comparison to values of Δ*H*, Δ*S* is expressed along with temperature (*T*Δ*S*), where *T* is 298.15 K. Experiments were performed in triplicate and standard errors are reported as standard deviations. Control experiments with the inverted direction of titration revealed no significant differences.

### Optical melting

Samples were prepared by diluting stock solutions of HPLC-purified oligonucleotides into buffers containing 200 mM NaHEPES, pH 7.5 and 100 mM of NaCl. Oligonucleotide concentrations were verified by UV absorbance at 260 nm using extinction coefficients predicted by primary sequence. All samples were heated to 98°C for 3 min and subsequently cooled to room temperature for 1 h before data were collected. Experimental RNA concentrations ranged between 200–6.75 μM and thermal melting curves were collected using an Agilent Cary 60 UV-Vis. Absorbance was recorded at 260 nm as the temperature was ramped between 4°C and 89°C at a rate of 1°C min^−1^. All data were collected in duplicate. Values of *T*_m_ were obtained by directly fitting the resulting melting curves to a double baseline, two-state model (29). These values were used to construct plots of *T*_m_^−1^ versus ln(*C*_T_/4), where *C*_T_ is the total RNA concentration. A linear least-squares fit was applied to the resulting plots and the van ′t Hoff Δ*H* and Δ*S* values were calculated from the slopes and intercepts according to the relationship:

\begin{equation*} \frac{1}{{T_M }} = \frac{R}{{\Delta H}}ln(C_T /4) + \frac{{\Delta S}}{{\Delta H}} \end{equation*}

Values of Δ*G* were calculated according to the relationship: Δ*G* = Δ*H* –*T*Δ*S*, where *T* is temperature in K. Errors on the reported values of *T*_m_ correspond to half the difference between replicates. In the case of the **U_c_:U_c_** homodimer, *C*_T_/4 was replaced by *C*_T_.

### Circular dichroism spectroscopy of ssRNA

Samples were prepared by diluting stock solutions of HPLC-purified nucleotides to 100 μM in buffer containing 200 mM NaHEPES, pH 7.5 and 100 mM of NaCl. Nucleotide concentrations were verified by UV absorbance at 260 nm using extinction coefficients predicted by primary sequence. RNA samples were heated to 98°C for 3 min and subsequently cooled to room temperature for 1 h before data were collected. CD spectra were collected using an Aviv Instruments CD Spectrometer Model 202.

### ^1^H NMR spectroscopy

NMR samples were prepared by dissolving nucleosides to a concentration of 10 mM in ^2^H_2_O at pH 7.5. ^1^H NMR spectra were collected at 25°C using a Varian 400 MHz NMR spectrometer (Oxford AS-400) equipped with a Varian 5 mm broadband PFG (z-gradient) probe. The data were analyzed using the Mnova NMR software suite. The 9 parameter parameterization (30) mode of the Matlab Pseudorotation GUI (31) was used to analyze the vicinal coupling constants of the ^1^H NMR spectra to afford the fractional populations of C3′-*endo* and C2′-*endo* states.

### Molecular dynamics simulations

MD simulations were performed using the program NAMD 2.9 (32) with the CHARM27 parameter set modified to include parameters for s^2^U (33). All simulations used periodic boundary conditions with Langevin dynamics and a Langevin piston (34) to maintain the temperature at 298.15 K (with a damping constant of 5 ps^−1^) and the pressure at 1 atm. The cutoff distance was 16 Å and the Particle Mesh Ewald Method (35) grid density was 1 Å^−3^.

The structures of nucleosides (U and s^2^U) were solvated in water boxes with periodic boundary conditions in VMD 1.9.1 using standard parameters. The resulting structures were minimized over 10 000 steps of 1 fs each. Unrestrained simulations were performed on each structure. Dihedral phase angles were calculated from the resulting trajectory files using the formula of Cremer and Pople (36). To increase sampling, 5 replica runs were performed with different minimized structures and initial velocities. Nucleosides were equilibrated for 10 ns followed by 50 ns production runs.

### Umbrella sampling

Umbrella sampling calculations were performed on nucleosides with a harmonic restraint centered on the dihedral angle describing the rotation of the nucleobase relative to the sugar (O4′-C1′-N1-C2, or the ‘chi’ angle), varying successively from 0^o^ to 360^o^ every 10^o^ with a force constant of 0.007 kcal·mol^−1^deg^−2^. Each simulation was 400 ps and five replicates were performed for both U and s^2^U. The free energy landscape was calculated using the Weighted Histogram Analysis Method (WHAM) (37).

### Desolvation penalty investigations

Crippen's Fragmentation estimations of LogP were generated in ChemBioDraw Ultra 13.0. Values of LogP were empirically determined by dissolving samples in 200 μl of un-ionized water at 25, 5 and 1.25 mM (verified by UV absorbance) before adding 200 μl of 1-octanol and vigorously vortexing the biphasic solutions before allowing them to separate overnight. LogP was calculated as the log of [solute]_1-octanol_/[solute]_water_ (38).

Desolvation energies were calculated using the General Born Ion model in the Adaptive Poisson-Boltzmann Solver (APBS) suite of software (39). First, PDB files were converted to PQR files using the PDB2PQR server version 1.8 with the CHARMM27 force field and the protonation states assigned at pH 7.0. Calculations were then performed using the APBS version 1.4 executable. The input file for this calculation appears in the supporting information.

## RESULTS

### Isothermal titration calorimetry (ITC) studies and thermal denaturation of ssRNA

**Figure 3.**
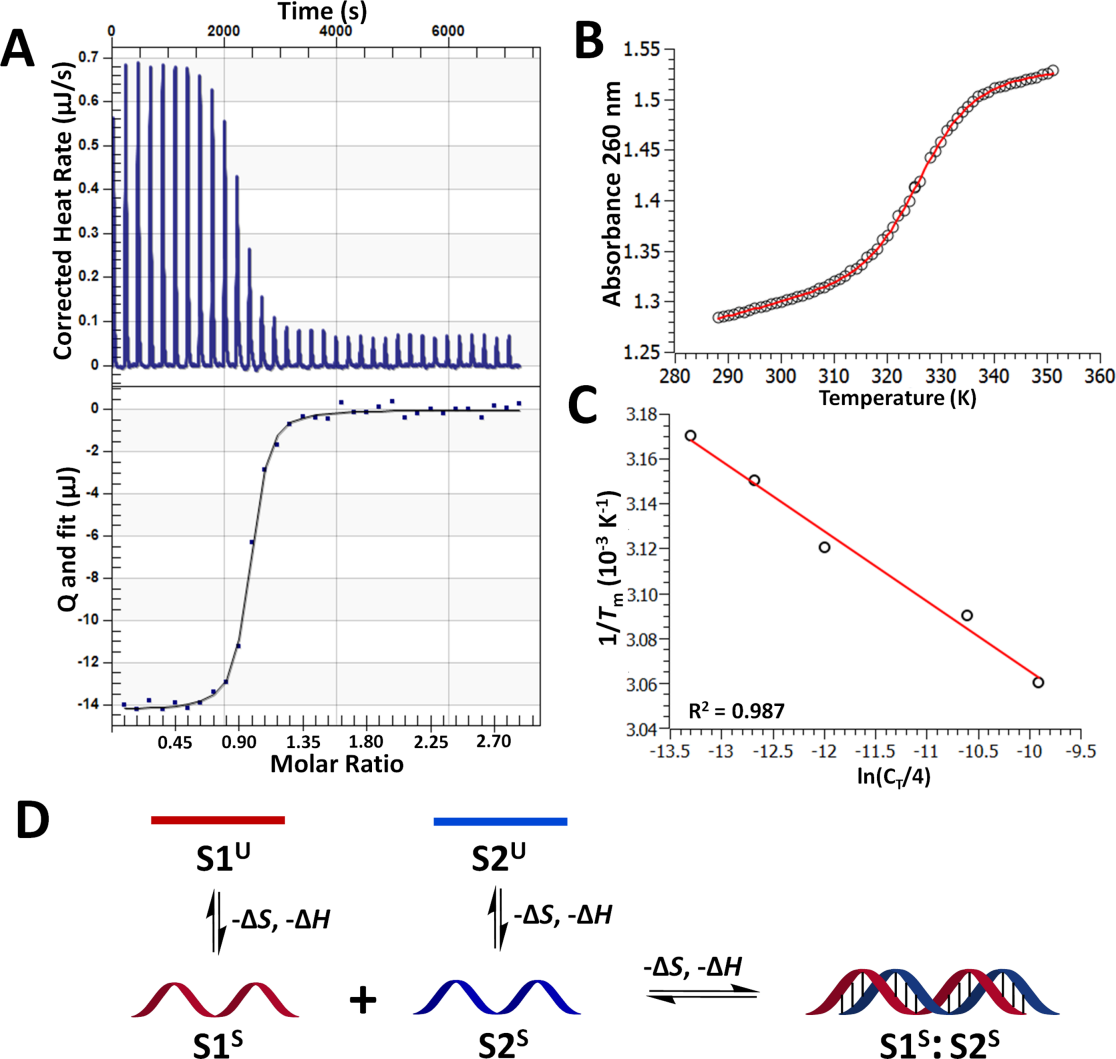
Experimental and mechanistic thermodynamic evaluations of duplex formation. (**A**) Representative ITC data measuring the thermodynamics of RNA duplex formation of **s^2^U:U_c_**. The s^2^U-containing ssRNA (100 μM) was titrated into a solution containing its complement (10 μM) in 200 mM NaHEPES, pH 7.5, 100 mM NaCl at 25°C. Top: Raw curve of power versus time. Bottom: Fit of integrated peak areas (heat released per injection) yielding the thermodynamic parameters presented in Table 1. Isotherms for the remaining duplexes appear in Supplementary Figure S1; (**B**) Melting curve (black circles) collected during the thermal denaturation of 200 μM **U:A** in 100 mM NaCl and 200 mM NaHEPES, pH 7.5 and the double-baseline, two-state fitted model (red line). Melting curves for the remaining duplexes appear in Supplementary Figure S2; (**C**) Linear least-squared fit (red line) of a van ′t Hoff plot of inverse melting temperatures (*T*_m_^−1^) collected from optical melts at different total oligonucleotide concentrations (*C*_T_) of **U:A**. Additional fits appear in Supplementary Figure S3; (**D**) Mechanistic scheme depicting the thermodynamic steps of duplex formation, beginning with the transition of single strands from an unstacked (S1^U^ and S2^U^) to a stacked (S1^S^ and S2^S^) state before the formation of the duplex (S1^S^:S2^S^). Both the stacking of the single strands and the duplex formation steps are expected to result in the release of heat and a negative change in entropy.

We evaluated the thermodynamic contributions of s^2^U toward RNA duplex stability by ITC. In all our experiments, a known amount of a single-stranded RNA is titrated into a solution containing a known amount of its complement or single mismatch, and the heat released upon binding is measured. A series of such additions is plotted to yield a curve of heat released as a function of titration progress (Figure 3A). All titration experiments were carried out in 200 mM NaHEPES, pH 7.5, 100 mM NaCl at 25°C.

It is important to consider that the presence of stable homodimers formed from the ssRNA in either the cell or the syringe during an ITC titration will decrease the observed favorability of the Δ*G* of duplex formation and will result in less favorable observed values of Δ*H*. We first carried out a series of variable concentration thermal denaturation experiments on solutions containing only the U, s^2^U, A or U_c_ strands, respectively, in order to evaluate this possibility. Thermal denaturation studies performed on the single strand U_c_ using standard UV absorbance methods indicate that significant homodimerization (formation of **U_c_:U_c_**) does occur at concentrations relevant to our ITC studies (Supplementary Figure S5). The resulting melting curves of U_c_ resemble those of typical double-stranded melting profiles, and the *T*_m_^−1^ values scale linearly with the natural log of concentration, an observation which is consistent with the formation of stable **U_c_:U_c_** homodimers. We propose that this homodimer is stabilized by two Watson–Crick G:C and four G:U wobble pairs. The thermodynamic parameters, namely the changes in enthalpy (Δ*H* = −67.6 kcal mol^−1^) and the equilibrium constant (*K*_d_ = 6.5 μM), obtained from the van 't Hoff analysis of this melting data were used in a multistep binding model in order to obtain the true values of Δ*G*_ITC_ and Δ*H*_ITC_ for **U:U_c_** and **s^2^U:U_c_** duplex formation, the derivation of which we explain below in brief.

In contrast, thermal denaturation studies of the other three ssRNAs (U, A and s^2^U) produced curves of a far less sigmoidal nature not suitable for the reliable calculation of *T*_m_ (Supplementary Figure S4). This observation is consistent with only weak intermolecular associations, if any. Although we are unable to quantify the *T*_m_ values for these melting processes, we can gain further insight into whether the observed process is inter- or intramolecular by normalizing the melting curves obtained at different concentrations and overlaying them on a single graph. For an intramolecular association, the curves theoretically should overlap completely, while for an intermolecular interaction, the midpoint of the curves should shift monotonically with respect to changes in concentration, indicating a change in melting temperature. The curves for U and s^2^U, respectively, overlap almost completely (Supplementary Figure S4 A and B) over the range of concentrations employed after normalizing the absorbance by concentration, suggesting that the melting behavior of these single strands arise primarily from an intramolecular process. In the case of the A strand, the melting behavior is significantly different. In particular, there is a concentration-dependent shift of the normalized A260 value at 0.75 (approximately the vertical midpoint) from higher temperature to lower temperature as the concentration of A is decreased (Supplementary Figure S4E). This shift points to an intermolecular interaction, likely the formation of a more weakly bound homodimer, in comparison to that of the U_c_ strand with the same number of G:C Watson–Crick but only two G:U wobble pairs. The presence of both the **U_c_:U_c_** and **A:A** dimers in solution during the ITC experiments will cause a decrease in the favorability of both the observed values of Δ*H*_ITC_ and Δ*G*_ITC_ to varying degrees.

Having an understanding of the single-stranded RNA dynamics, we went ahead and carried out the four sets of ITC experiments for **s^2^U:A**, **U:A**, **s^2^U:U_c_** and **U:U_c_**. The observed values of Δ*G*_ITC_ for **U:A** and **s^2^U:A** are −10.0 and −10.5 kcal mol^−1^, while the observed values of Δ*H*_ITC_ are −47.7 and −45.5 kcal mol^−1^, respectively. These measurements lead to calculated values of *T*Δ*S*_ITC_ (25°C) of −37.7 and −35.0 kcal mol^−1^. At face value, these ITC results indicate that the presence of s^2^U results in enhanced duplex stability. The greater duplex stability of **s^2^U:A** compared to **U:A** results from a reduced entropic penalty, the effect of which is partially compensated by a reduction in heat released upon binding. In the case of the duplexes containing a single mismatch, the observed values of Δ*G*_ITC_ for **U:U_c_** and **s^2^U:U_c_** are –7.24 and –8.65 kcal mol^−1^, while the observed values of Δ*H*_ITC_ are –9.14 and –14.7 kcal mol^−1^, respectively. Note that these uncorrected observed values of Δ*H*_ITC_ are drastically more positive in comparison to the values observed for **s^2^U:A** and **U:A** (Table 1). These dramatically less favorable observed values of Δ*H*_ITC_ are most likely the result of **U_c_:U_c_** homodimer formation.

**Table 1.** The Thermodynamic Parameters of RNA Duplex Formation by Isothermal Titration Calorimetry and Thermal Denaturation

entry	duplex	base pair	*K*_d_^a^ (nM)	Δ*H*_ITC_^a^ (kcal mol^−1^)	*T*Δ*S*_ITC_^b^ (kcal mol^−1^)	Δ*G*_ITC_^c^ (kcal mol^−1^)	Δ*G*_vH_^d^ (kcal mol^−1^)	*T_m_*^e^ (°C)
1	5′-uagc**U**cc-3′	U:A	45.2(5)	–47.7(2)	–37.7(3)	–10.0(1)	–11.3	53.6(1)
	3′-aucg**A**gg-5′						(–12.0)	
2	5′-uagc**s^2^U**cc-3′	s^2^U:A	21.1(2)	–45.5(2)	–35.0(2)	–10.5(1)	–12.8	64.7(4)
	3′-aucg**A**gg-5′							
3	5′-uagc**U**cc-3′	U:U	1010(120)	–64.3(1)	–56.1(1)	–8.18(1)	–7.6	35.5(1)
	3′-aucg**U_c_**gg-5′						(–7.7)	
4	5′-uagc**s^2^U**cc-3′	s^2^U:U	232(26)	–55.0(1)	–46.0(1)	–9.05(1)	–8.4	44.3(7)
	3′-aucg**U_c_**gg-5′							

^a^Δ*H*_ITC_ and *K*_d_ values were evaluated directly from ITC titration data for **U:A** and **s^2^U:A** using a least-squares nonlinear regression analysis described previously (28) and for **U:U_c_** and **s^2^U:U_c_** using a model designed to compensate for significant self interactions observed by thermal melting for 3′-aucg**U**gg-5′ (for details of this model and stoichiometry values (n), see the SI).

^b^*T*Δ*S*_ITC_ was calculated according to *RT* ln(*K*_d_) = Δ*H*_ITC_ –*T*Δ*S*_ITC_ where *R* is the gas constant and *T* is temperature.

^c^Δ*G*_ITC_ was calculated using values of *K*_d_ from the ITC data according to Δ*G*_ITC_ = *RT* ln(*K*_d_). All ITC titrations were performed in triplicate at 25°C.

^d^Δ*G*_vH_ was calculated from thermal denaturation data collected in duplicate and according to Δ*G*_vH_ = Δ*H*_vH_ –*T*Δ*S*_vH_, where values of Δ*H*_vH_ and Δ*S*_vH_ (Supplementary Table S1) were derived from linear fits of van ′t Hoff plots of *T*_m_^−1^ versus ln(*C*_T_/4) where *C*_T_ is the total oligonucleotide concentration. The *R*^2^ values of the plots for entries 1, 3 and 4 are approximately 0.99. The *R*^2^ value of entry 2 is slightly lower at 0.94 (Supplementary Figure S3), possibly due to a premelting transition that persists at all oligonucleotide concentrations tested here. Values in parentheses are predictions based upon a nearest neighbor model at 1 M NaCl (40).

^e^*T*_m_ was calculated from a double baseline, two-state fit of raw optimal melt data at a *C*_T_ of 200 μM (see Materials and Methods for additional details). *T*_m_ values are averages and the errors correspond to half of the differences between the measured values.

Correcting for the effect of homodimerization on the thermodynamic parameters obtained by ITC demanded the use of a multistep mechanistic model of duplex formation. A complete derivation and description of this model can be found in the SI. The model is based on a mechanism in which the **U:U_c_** (or **s^2^U:U_c_**) duplex can only form from the fraction of **U_c_** not bound up in the **U_c_:U_c_** homodimer. Explicitly, the following multistep equilibrium is assumed:

(1) \begin{equation*} {^1{/}_2}[{\rm U_c:U_c}]\mathop{\rightleftharpoons}^L [{\rm U_c}]\mathop{\rightleftharpoons}^{K[{\rm U}]}[{\rm U:U_c}] \end{equation*}

where *L* is the equilibrium constant governing the dimerization of U_c_ and *K* is that for **U:U_c_**. The value of *L* was measured independently from the single-strand melts described earlier. From this multistep equilibrium, we show that the value of the observed binding constant *K*_OBS_ when measured by fitting to a single-step binding isotherm becomes related to *K* by the following expression:

(2) \begin{equation*} K_{{\rm OBS}} = \frac{K}{{1 + 2L\left[ {{\rm U}_{\rm c} } \right]}} \end{equation*}

by knowing the value of *L* and the concentration of free U_c_ in the cell, we can calculate the true value of *K*.

From this multistep equilibrium, how the observed enthalpy Δ*H*_OBS_ is affected by the presence of the **U_c_:U_c_** homodimers can be described. Specifically, the observed change in enthalpy is equal (28) to the change in mole fractions of both the **U_c_:U_c_** dimer and the **U:U_c_** duplex with respect to the beginning and end of the titration, with each term multiplied by their respective molar enthalpies. This formalism leads to the following result:

(3) \begin{equation*} \Delta H_{{\rm OBS}} = \Delta H_{{\rm U}:{\rm U}_{\rm c} } - \Delta H_{{\rm U}_{\rm c} :{\rm U}_{\rm c} } \left( {\frac{{2L\left[ {{\rm U}_{\rm c} } \right]}}{{1 + 2L\left[ {{\rm U}_{\rm c} } \right]}}} \right) \end{equation*}

where [U_c_] is the initial concentration of free U_c_ at the beginning of the titration.

With this model in hand, we can correct the observed values of Δ*G*_ITC_ and Δ*H*_ITC_ for the **U:U_c_** and **s^2^U:U_c_** duplexes. The values of Δ*G*_OBS_ are only slightly affected, becoming −8.18 and −9.05 kcal mol^−1^, respectively. The values of Δ*H*, however, undergo much larger changes to −64.3 and −55.0 kcal mol^−1^ (Table 1). Like in the case of the **s^2^U:A** and **U:A** duplexes, 2-thiolation again results in greater duplex stability, even in the context of a mismatch. Duplex formation is more favorable by 0.5 kcal mol^−1^ for **s^2^U:A** and by 0.9 kcal mol^−1^ for **s^2^U:U_c_**, compared to their native counterparts, **U:A** and **U:U_c_**. These values are consistent with previously reported optical melting studies of these (27) and other s^2^U-containing RNAs (21). The greater duplex stability of **s^2^U:U_c_** compared to **U:U_c_** resulted from a reduced entropic penalty (*T*Δ*S*_ITC_ at 25°C = –46.0 and –56.1 kcal mol^−1^, respectively), the effect of which is partially diminished by a reduction in heat released upon binding (Δ*H*_ITC_ = –55.0 and –64.3 kcal mol^−1^, respectively). These results indicate that 2-thiolation increases the favorability of duplex formation for both **s^2^U:A** and **s^2^U:U_c_** and that these effects may both result from analogous mechanisms.

### Thermal denaturation of Duplex RNA

To increase confidence in the thermodynamic parameters derived from ITC, we measured the melting temperatures (*T*_m_) of the four dsRNAs under the same conditions as the ITC experiments (100 mM NaCl and 200 mM NaHEPES at pH 7.5) over a range of RNA concentrations using variable temperature UV absorbance methods (Table 1) (41). The resulting melting curves were fit with a double-baseline, two-state model (42) (Figure 3B) and the values of *T*_m_^−1^ were plotted as functions of ln(*C*_T_/4), where *C*_T_ is the total RNA concentration (Figure 3C). The slope and intercept of the resulting van 't Hoff plots afford estimations of Δ*G*_vH_, Δ*H*_vH_ and Δ*S*_vH_ for duplex formation (29). The *T*_m_ and Δ*G*_vH_ values appear in Table 1.

The values of *T*_m_ for the s^2^U-containing duplexes exceed those of their native counterparts by 11.1°C for **s^2^U:A** and 8.8°C for **s^2^U:U_c_** at 200 μM total oligonucleotide concentration. These results are consistent with those we reported previously for identical sequences (27) as well as literature values for similar sequences (21) and confirm that s^2^U enhances the thermal stability of dsRNA. We also observed significant premelting transitions in the case of **s^2^U:A** (Supplementary Figure S2), that may arise from changes in secondary structure or torsional rigidity, the fraying of terminal basepairs, or other structural changes (43) and can complicate van 't Hoff analyses. Despite these complications, the values of Δ*G*_vH_, Δ*H*_vH_ and Δ*S*_vH_ obtained from our thermal denaturation studies exhibit the same trend as those obtained from ITC and, in each case, agree that 2-thiolation enhances the stability of dsRNA. The differences in Δ*G*_vH_, Δ*H*_vH_ and Δ*S*_vH_ for **U:A** in comparison to **s^2^U:A** are consistent with similar sequences studied by Kierzek *et al*. (21) (Supplementary Table S1). The values of Δ*G*_vH_, Δ*H*_vH_ and Δ*S*_vH_ for **U:A** and for **U:U_c_** also conform to the predictions of nearest-neighbor models, the latter of which takes into consideration the single, internal U:U mismatch (40).

### Circular dichroism studies of single-stranded RNA

**Figure 4.**
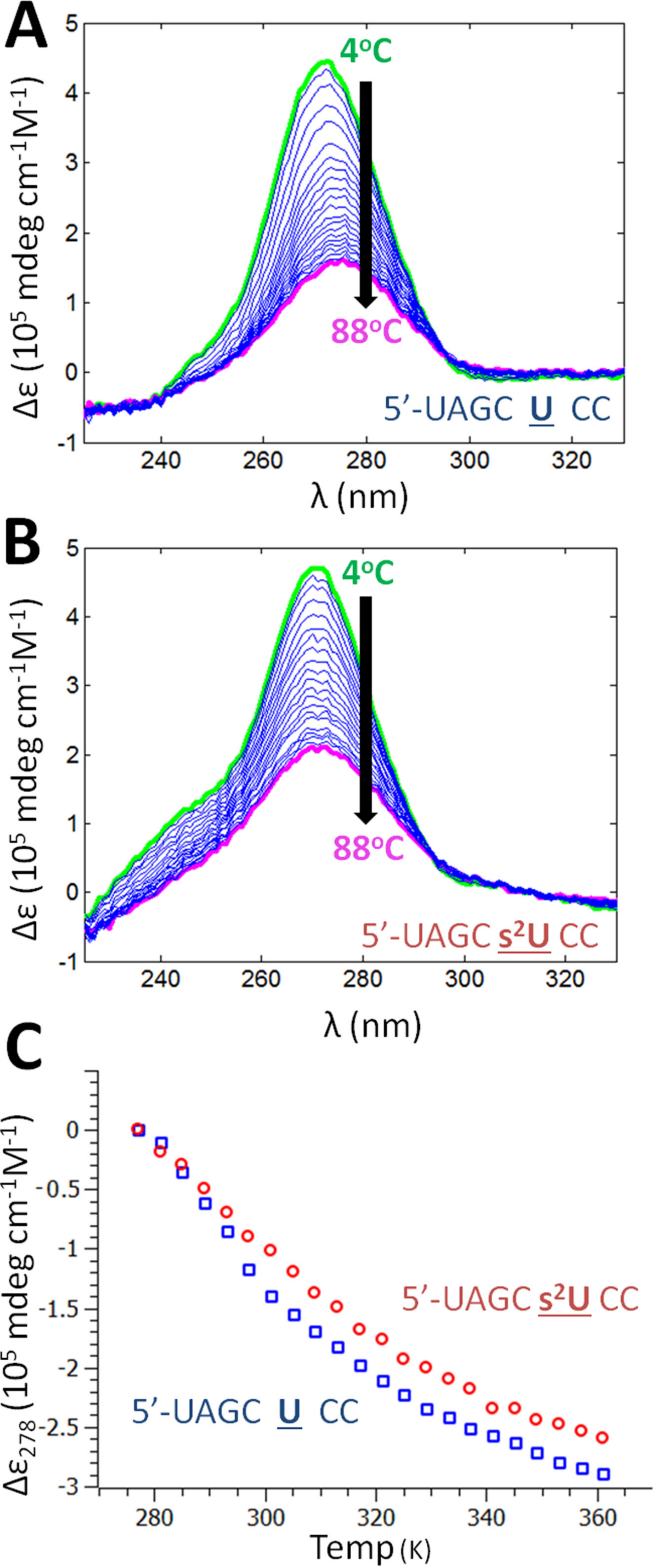
Circular dichroism studies of single-stranded RNA. (**A**) Overlapped spectra obtained by UV-CD spectroscopy of 5′-uagc**U**cc-3′ at temperatures ranging from 4°C to 88°C using 50 μM oligonucleotide in buffer containing 100 mM NaCl and 200 mM NaHEPES at pH 7.5; (**B**) The identical experiment performed using 5′-uagc**s^2^U**cc-3′; (**C**) Normalized UV-CD signal of the s^2^U-containing ssRNA and its native counterpart at 278 nm.

CD spectra afford information about the chirality and secondary structure of biomolecules, including RNA. The CD spectra of both dsRNA and ssRNA feature a prominent maximum near 280 nm. In both cases, this peak recedes toward the baseline as secondary structure is disrupted by thermal melting or other processes such as chemical denaturation (44). We performed CD melts on the s^2^U-containing ssRNA and its native counterpart. These experiments were performed under identical conditions to the UV melts. As expected, we observed an absorption maximum near 280 nm which diminished as the temperature was ramped from 4°C to 88°C (Figure 4A). The same trend was observed for both of the ssRNAs, but the CD signal of the s^2^U-containing ssRNA at 4°C exhibits a prominent feature near 240 nm and a negative peak near 330 nm (Figure 4B). The maximum near 280 nm was greater for the s^2^U-containing ssRNA over the entire temperature range tested. The signal for the s^2^U-containing ssRNA recedes at a lesser rate with respect to increasing temperature compared to its native counterpart over the temperature range examined here (Figure 4C). CD signals for U and s^2^U nucleosides were found to be nearly constant over a similar temperature range (Supplementary Figure S4), suggesting that the helical conformations of the single strands are responsible for the observations described here. For a discussion of the denaturation of the single strands observed by UV, see the SI.

### ^1^H NMR studies of U and s^2^U nucleosides

The structural effects of s^2^U on ssRNA potentially originate from the conformational bias of the nucleoside itself. Any given nucleoside will preferentially adopt a sugar pucker conformation in solution that can be described as a distribution between C2′-*endo* and C3′-*endo* states (Figure 2). The ribose groups of structured RNA normally prefer C3′-*endo* conformation (A-form) as it prevents steric clashes between 2′-OH groups and the backbone and results in maximal base stacking interactions (45). It has also been demonstrated that nonenzymatic, template-directed RNA copying is most rapid when all three components—template, primer and activated monomer—are in the A-form conformation (46). To evaluate the effect of 2-thiolation on nucleoside sugar-pucker conformation, we evaluated the NMR spectra of U and s^2^U nucleosides at 25°C. Although this has been previously investigated, we decided to revisit this approach using a greater magnetic field strength (400 versus 270 MHz) and more precise temperature control, enabling a more reliable assessment of coupling constants. We also employed a more sophisticated version of the Karplus equation, affording more accurate estimations of the fractional populations. The fractional populations of the nucleoside in the C2′-*endo* and C3′-*endo* forms were calculated from the vicinal spin-coupling constants using the Matlab Pseudorotation GUI (31), which utilizes Diez's modification of the Karplus equation (47). The quotients of the fractional populations at 25°C give rise to equilibrium constants, enabling the determination of the free energy change separating conformational states using the relationship Δ*G* = –*RT* ln(*K*_eq_), where *R* is the gas constant and *T* is the temperature in K. This method confirmed previous results (48) demonstrating that U and s^2^U nucleosides both preferentially adopt C3′-*endo* conformations in solution, with the s^2^U nucleoside favoring C3′-*endo* to a greater extent. Our results indicate that the fractional population of nucleoside in the C3′-*endo* conformation was significantly higher for s^2^U than U (80% and 54%, respectively, Table 2) at 25°C. The free energy difference between C2′-*endo* and C3′-*endo* at 25°C is approximately 0.7 kcal mol^−1^ greater for s^2^U than U (Table 2).

**Figure 5.**
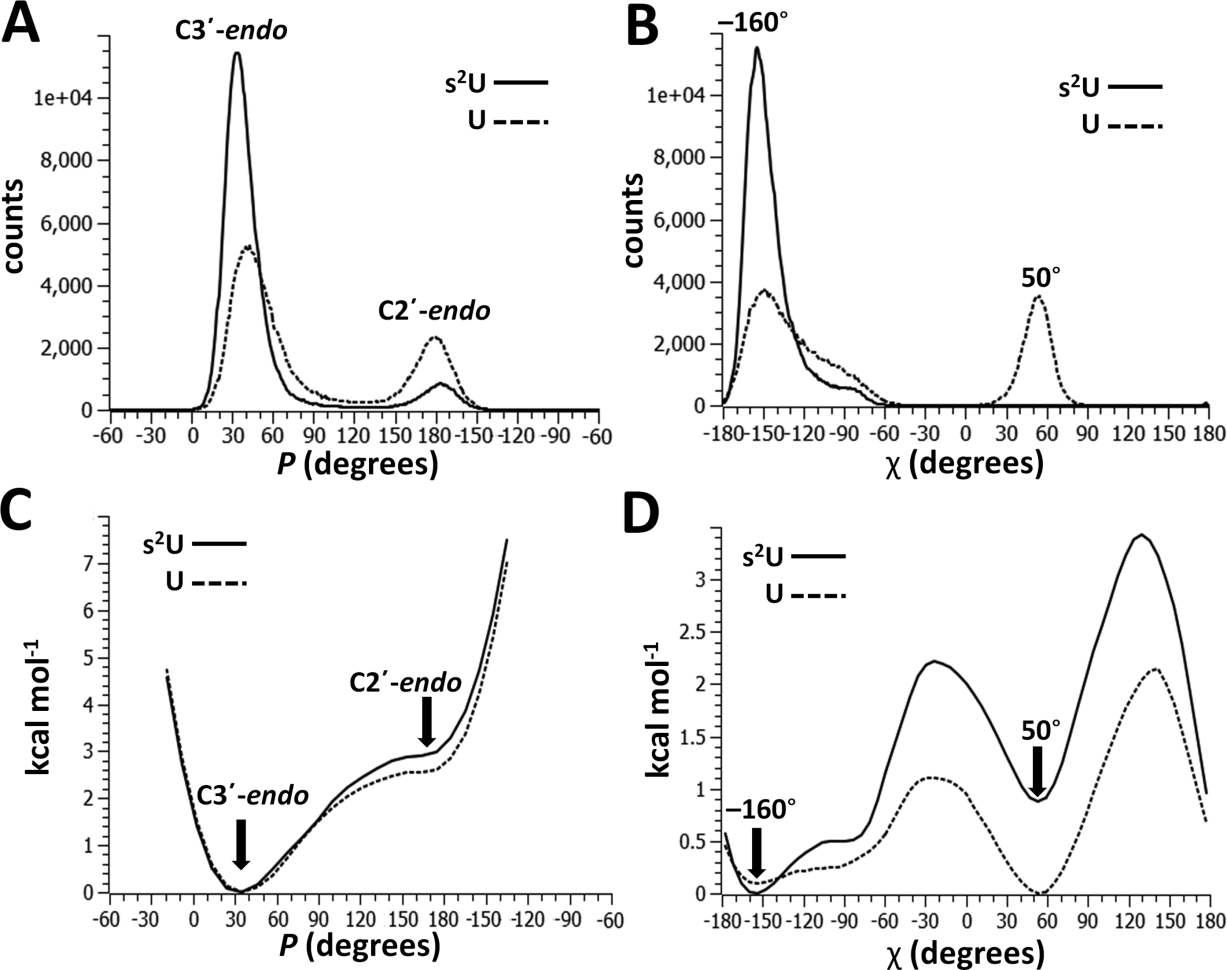
MD simulations and umbrella sampling of U and s^2^U nucleosides at 25°C. (**A**) Conformation histogram of the pseudorotation phase angle (*P*). s^2^U spends a larger proportion of time in the preferred C3′-*endo* conformation; (**B**) Conformation histogram of chi angle (χ). s^2^U almost exclusively occupies a χ of ∼–160° while U spends a significant portion of the simulation occupying a χ of ∼50°; (**C**) Free energy landscape for *P*. Energy minima appear at values corresponding to C3′-*endo and* C2′-*endo* conformations. The barrier separating these conformations is larger for s^2^U; (**D**) Free energy landscape for χ. Energy minima appear at values corresponding to ∼–160° and ∼50°. The barrier separating these conformations is, again, larger for s^2^U.

**Table 2.** Nucleoside Conformation and Solvation Data

Nucleoside	Fractional population^a^ C3′-*endo*	Fractional population^a^ χ (–160°)	Δ*G*^b^ C3′-*endo* (kcal mol^−1^)	Δ*G^b^* χ (–160°) (kcal mol^−1^)	Desolvation Free Energy (kcal mol^−1^)^c^	LogP^d^ (calculated)	LogP^e^ (measured)
U	54%	NA	–0.09	NA	32.1	–2.28	–1.78(5)
	(71%)	(67%)	(–0.54)	(–0.41)			
s^2^U	80%	NA	–0.81	NA	28.7	–1.29	–1.17(7)
	(92%)	(>99%)	(–1.43)	(NA)^f^			

^a^The fractional populations of nucleoside in the C2′-*endo* and C3′-*endo* form were calculated from ^1^H NMR vicinal spin coupling constants using Diez's modification of the Karplus equation. Values in parentheses are from MD simulations (Figure 5).

^b^Calculated using the relationship Δ*G* = –*RT*ln(*K*_eq_), where *R* is the gas constant, *T* is the temperature in K, and *K*_eq_ is based upon the fractional populations of each conformation. Values in parentheses are from MD simulations.

^c^Calculated using the APBS software suite (see Materials and Methods).

^d^Calculated using Crippen's Fragmentation, a structure-based estimation.

^e^Determined by partitioning the nucleoside of interest between deionized water and 1-octanol. The error for LogP (measured) is the standard deviation from separate experiments.

^f^There is no observable population in the C2′-*endo* conformation, invalidating the calculation of Δ*G*.

### Molecular dynamics (MD) simulations and umbrella sampling

Simulating molecular dynamics enables the investigation of higher energy conformations and the intermediate states separating them. To assess the effect of 2-thiolation on conformational dynamics, we performed MD simulations and umbrella sampling on U and s^2^U nucleosides.

Concisely and accurately describing RNA conformations requires a set of carefully chosen collective variables (CVs) that distinguish all significant conformers. The CVs chosen here are the phase angle of pseudorotation (*P*) and the chi torsion angle (χ) (45). *P* is a convenient descriptor of nucleoside sugar pucker; values of ∼30° and ∼180° correspond to C3′-*endo* and C2′-*endo* conformations, respectively. χ characterizes the relative orientation of base and sugar. χ values of approximately –160° are most often observed in structured RNA and correspond to maximal base stacking interactions.

We performed five replicate MD simulations of s^2^U and U over 50 ns at 25°C with explicit water. The observations of the simulations match those of the NMR studies: s^2^U occupied the C3′-*endo* conformation for ∼90% of the simulation. This preference was much stronger than that of U which spent only ∼70% of the simulation in the C3′-*endo* (Figure 5). These fractional populations indicate that the free energy difference separating C3′-*endo* and C2′-*endo* conformations is roughly 0.9 kcal mol^−1^, a value almost identical to that calculated from ^1^H NMR (Table 2). The MD simulations also predict that 2-thiolation has a significant effect on χ. The χ value of s^2^U remained constant at –160° while U alternated between the conformation corresponding to a χ value of –160° and a second conformation corresponding to 50°. The χ value of 50° appears to be a low energy conformation for the free nucleoside, but it is unlikely to result in optimal base–base stacking in the structured RNA (Figure 2E).

To further investigate the high energy conformations not sampled by MD and to probe the effect of 2-thiolation on the free energy landscapes of *P* and χ, we proceeded to perform umbrella sampling on the nucleosides. The resulting free energy landscapes (Figure 5C,D) are consistent with the results of the MD simulations. The free energy landscapes of *P* for both nucleosides feature a global minimum at ∼30°, corresponding to the C3′-*endo* conformation, and a local minimum near ∼180°, corresponding to the C2′-*endo* conformation. The free energy barrier between these states is ∼0.5 kcal mol^−1^ larger for s^2^U than U. Values of *P* ‘left’ of –30° and ‘right’ of –120° were not sampled as these regions correspond to very high energy conformations not observable by this simulation.

The free energy landscapes of χ for both nucleosides feature prominent minima near –160° and 50°, corresponding to the values of χ observed by MD. For s^2^U, the global minimum at –160° is ∼1 kcal mol^−1^ lower in energy than the local minimum at 50°. However, for U, the energy of the same two minima is almost indistinguishable.

### Evaluation of the desolvation penalty

Solvent-oriented H-bond acceptors are often desolvated during duplex formation (25). The greater the strength of the H-bond acceptor, the greater the free energy penalty for this process (49). Removing the H-bond acceptor entirely or substituting it with a weaker acceptor, such as S, has been shown to partially alleviate this penalty (25). In order to evaluate the effect of desolvation in our system, we measured the difference in partition coefficients (LogP) between s^2^U and U nucleosides and compared the measured differences to theoretical predictions (Table 2). The values of LogP for s^2^U and U, measured directly by partitioning s^2^U and U nucleosides in a biphasic system of deionized water and octanol, were found to be −1.17 and −1.78, respectively. We employed Crippen's Fragmentation (50), a structure-activity relationship-based approach, to afford theoretical partition coefficient estimations for comparison. These theoretical values of LogP for s^2^U and U were calculated to be −1.29 and −2.28, respectively, numbers that agree well with the experimentally determined values. Next, we computationally evaluated the Born Ion Solvation Energy (51) using the APBS software suite, and this level of theory agreed that desolvation penalty for s^2^U is 3.4 kcal mol^−1^ less than for U (Table 2).

## DISCUSSION AND CONCLUSION

Nearly all nucleic acid complexations, including the formation of duplexes, triplexes and tetraplexes, are associated with overall favorable changes in enthalpy (–Δ*H*) and unfavorable changes of entropy (–Δ*S*) (52). For short RNAs, duplex formation can be conceptualized thermodynamically as a two-step process (Figure 3D) (29). The single strands must first proceed from an unstacked random coil to a stacked helical conformation before forming the duplex. A favorable change in enthalpy is associated with both steps: heat is released as the bases stack during the ordering of the single strands; and next, as H-bonds are formed during duplex formation. The enthalpic favorability of this process is partially countered by a desolvation penalty (53), but overall, each step can be expected to be associated with a negative change in enthalpy. Hybridization also results in an overall increase in order, as reflected by the negative sign of Δ*S* associated with RNA duplex formation. The ITC results for each duplex examined here conform to these expectations and the trends in duplex stability are consistent with those determined from the thermal denaturation data and with the predictions of a nearest neighbor analysis (Table 1), with one exception. The inability to quantitatively account for the possible homodimerization of the A strand has likely resulted in values of Δ*H*_ITC_ observed for **U:A** and **s^2^U:A** (−47.7 and −45.5 kcal mol^−1^, respectively) that are less negative than their true values. This reasoning likely explains why the observed values of Δ*H*_ITC_ are less favorable than their van 't Hoff counterparts. In addition, if a correction to the observed values could be applied, we hypothesize the values of Δ*H*_ITC_ for **s^2^U:A** and **U:A** would become more negative than the corrected values for **s^2^U:U_c_** and **U:U_c_**. Nevertheless, the main finding that 2-thiolation increases duplex stability by lessening the entropic cost of formation is not expected to change by such a correction, although the magnitude of the difference between the enthalpies of formation for **s^2^U:A** and **U:A** might.

Because Δ*H* and Δ*S* are related in part to the formation of H-bonds and stacking interactions during hybridization, mechanistic information can be obtained from the differences in these changes (ΔΔ*H* and ΔΔ*S*) resulting from 2-thiolation. The values of ΔΔ*H*_ITC_ and ΔΔ*S*_ITC_ are 2.2 kcal mol^−1^ and 9.1 cal mol^−1^ K^−1^, respectively, for **s^2^U:A** compared to **U:A** and 9.3 kcal mol^−1^ and 33.9 cal mol^−1^ K^−1^, respectively, for **s^2^U:U_c_** compared to **U:U_c_**. The source of this reduced entropic penalty is likely the contribution of 2-thiolation to the structural preorganization (26) of the ssRNA. Similar preorganizing effects have been implicated in biasing anticodon structure toward the A-form which has the effect of improving anticodon/codon interactions (54). This may partially explain the high conservation of s^2^U at the tRNA anticodon site.

The defining feature of a preorganized structure is the loss of some amount of its conformational flexibility (26), which is often assumed to result in smaller entropic penalties during binding (ex. hybridization) (52). By this reasoning, any change that shifts the equilibrium of a single stranded RNA toward the highly stacked and conformationally ordered state—*i.e*. single-strand preorganization—will result in a reduced entropic penalty for duplex formation. Recalling the definition of Gibbs free energy, Δ*G* = Δ*H* –*T*Δ*S*, a change that leads to enhanced preorganization of the ssRNA will most likely correspond to a positive ΔΔ*S*. A more preorganized single strand also requires less additional stacking to facilitate duplex formation, corresponding to less heat being released and a positive ΔΔ*H*. These differences are precisely what are observed by ITC when comparing duplex formation for the s^2^U-containing duplexes against their native counterparts (**s^2^U:A** vs **U:A** and **s^2^U:U_c_** vs **U:U_c_**) and is consistent with the results of Kumar (17). The results of the single-strand thermal denaturation experiments monitored by CD are consistent with the hypothesis that 2-thiolation leads to greater single-strand order between 4 and 88°C and greater resistance to melting, further supporting the hypothesis that 2-thiolation contributes to a more highly ordered single strand.

Further evidence for the hypothesis that s^2^U preorganizes the single strand can be found by comparing the crystal structures of **s^2^U:U_c_** and **U:U_c_** (Figure 2). The crystal structure of **U:U_c_** contains four duplexes with four distinct H-bond geometries at the site of the U:U base pair, two of which appear ‘weak’ based upon [N−H···O] bond lengths and angles (Figure 2C). These distinct geometries suggest that **U:U_c_** exists in solution in several conformations of comparable energy with disparate H-bond strengths. The crystal structure of **s^2^U:U_c_**, however, contains only a single duplex conformation with a single ‘strong’ H-bond geometry at the site of the s^2^U:U base pair, in which the S atom is directly involved as an H-bond acceptor. Based upon these structures, it appears that 2-thiolation favors a single strong H-bond geometry that resolves the duplex structure into a single conformation. This explanation is consistent with the observed thermodynamic parameters and is compatible with the hypothesis that 2-thiolation preorganizes the s^2^U-containing single strand.

To evaluate the hypothesis that 2-thiolation contributes to a stronger N3 H-bond due the increased acidity of the N3 imino proton, we repeated the determination of pKa values for both U and s^2^U nucleosides using pH titrations monitored by UV and NMR spectroscopies (Supplementary Figure S7). Both methods agree that the N3 imino proton of s^2^U is more acidic than that of U by ∼1 pKa unit. This observed difference likely results from the much lower pKa of an aryl thiol versus an aryl hydroxyl, which would stabilize the thiolate resonance structure of the conjugate base. Although the thermodynamic data collected here cannot be directly used to confirm a stronger N3 H-bond resulting from 2-thiolation, this hypothesis remains plausible.

We propose that the source of the observed single strand stabilization is, to a large degree, the effect of 2-thiolation on the nucleoside conformation. The ^1^H NMR results demonstrate that 2-thiolation stabilizes the preferred C3′-*endo* conformation of the nucleoside in solution. The free energy difference observed by NMR at the nucleoside scale is of similar magnitude to the difference observed for duplex formation by ITC (Table 1, all at 25°C). The NMR results are well complemented by MD and umbrella sampling efforts, both of which agree that 2-thiolation produces a greater preference for the C3′-*endo* conformation and a χ angle comparable to that observed in structured RNA. The predicted differences in free energy are similar to those observed by NMR, ITC and duplex thermal denaturation. Previous NMR studies (48) have suggested that 2-thiolation results in a steric interaction between the 2-thiocarbonyl and the bulky 2′-OH, potentially resulting in the heightened free energy barriers predicted here.

With respect to desolvation, the importance of this effect may be highly dependent on the duplex under consideration. The O2 atom of U5 in the crystal structure of **U:A** does not participate directly in a base pair H-bond but is situated within H-bond distance (2.9 Å, measured from the center of the S and O atoms) of a water oxygen atom (Supplementary Figure S8). In addition to possessing a highly similar overall structure to that of **U:A** (overlap RMSD of < 0.2 Å), the crystal structure of **s^2^U:A** shares both of these features—the S2 atom does not participate directly in a base pair H-bond but is situated within H-bond distance (3.4 and 3.5 Å) of two water oxygen atoms. These distances agree well with the known geometric characteristics of H-bonds involving S atoms (55). In this case, 2-thiolation does not appear to result in desolvation of the S atom and, therefore, we hypothesize that a reduced desolvation penalty for s^2^U is unlikely to contribute greatly toward the enhanced free energy of duplex formation for **s^2^U:A**.

In contrast, the S atom in the crystal structure of **s^2^U:U_c_** participates directly in a base pair H-bond and appears to be fully desolvated as no water oxygen atoms are resolvable within 4 Å from the S2 atom. The crystal structure resolution of **U:U_c_** is insufficient to determine whether or not the corresponding O2 atom is solvated. In the case of **s^2^U:U_c_**, it is at least plausible that a reduced desolvation penalty for s^2^U may contribute to the enhanced free energy of duplex formation.

s^2^U and other modified nucleobases may prove vital to improving the fidelity and rate of template-directed, nucleic acid replication systems, enzymatic or otherwise. The ubiquity and conservation of modified nucleobases strongly suggests that this chemistry has been commonly employed by nature to strengthen weak interactions and enhance accurate base–base recognition. From either a chemical biology or origins of life perspective, it is conceivable that the use of modified nucleobases may assist in the realization of a self-replicating RNA system. From a purely chemical perspective, it is impressive to consider the significant effect that a single, seemingly innocuous, atomic substitution can have on a large and complex system. By augmenting and informing ongoing structural and *in silico* studies of other modified nucleobases, this work may inform future studies of exotic nucleic acids and assist in their growing utility.

## Supplementary Material

SUPPLEMENTARY DATA
