# Supplementary material for: Thermodynamic insights into 2-thiouridine-enhanced RNA hybridization
Source: Nucleic Acids Res. 2015 Aug 3;43(16):7675–87. doi: 10.1093/nar/gkv761 (PMC4652770; doi:10.1093/nar/gkv761)
Supplement: SUPPLEMENTARY DATA [file supp_43_16_7675__index.html]

Thermodynamic insights into 2-thiouridine-enhanced RNA hybridization — Thermodynamic insights into 2-thiouridine-enhanced RNA hybridization — SUPPLEMENTARY DATA 

# Thermodynamic insights into 2-thiouridine-enhanced RNA hybridization

## SUPPLEMENTARY DATA

- SUPPLEMENTARY DATA
