# Supplementary material for: Thermodynamic insights into 2-thiouridine-enhanced RNA hybridization
Source: Nucleic Acids Res. 2015 Aug 3;43(16):7675–87. doi: 10.1093/nar/gkv761 (PMC4652770; doi:10.1093/nar/gkv761)
Supplement: SUPPLEMENTARY DATA [file supp_gkv761_nar-00951-r-2015-File002.docx]

Supporting Information

**Thermodynamic Insights into 2-Thiouridine-Enhanced**

**RNA Hybridization**

Aaron T. Larsen,^1^ Albert C. Fahrenbach,^1,2^ Jia Sheng,^3^ Julia Pian,^1^ and Jack W. Szostak^1,2^*

^1^Howard Hughes Medical Institute, Center for Computational and Integrative Biology, and Department of Molecular Biology, Simches Research Center, Massachusetts General Hospital, Boston, MA 02114

^2^Earth-Life Science Institute, Tokyo Institute of Technology, 2-12-1 Ookayama, Meguro-ku, Tokyo 152-8551, Japan

^3^ University at Albany, State University of New York, Department of Chemistry, The RNA Institute, 1400 Washington Avenue, Albany, NY 12222

CONTENTS

1) ITC Data and Analysis

2) Two-Strand Melting Curves and Analysis

3) Single-Strand Melting Curves and Analysis

4) pH Titrations

5) Derivation of the ITC Model

6) Crystal Structure Analysis

7) Calculations

1) ITC Data and Analysis


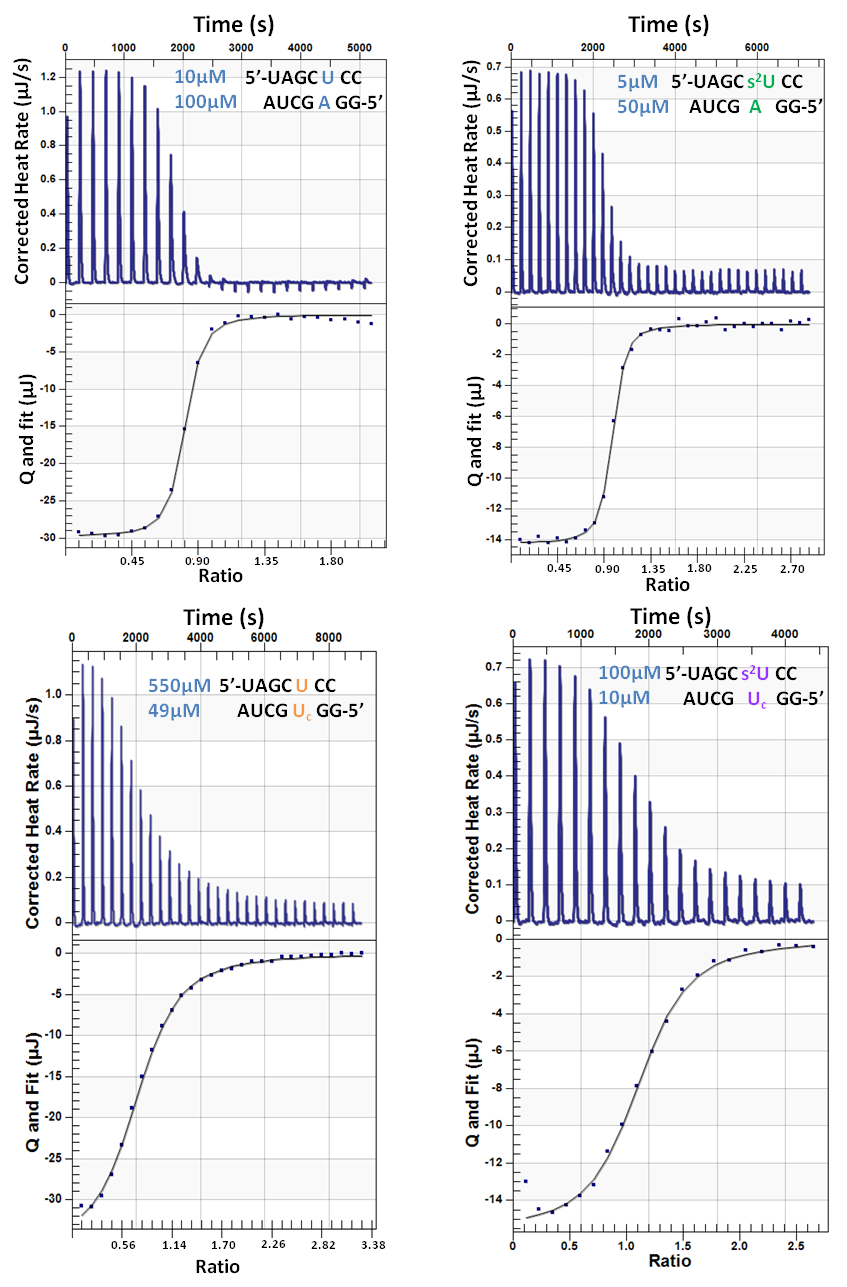


**Figure S1.** ITC data measuring the thermodynamics of RNA duplex formation at 25 °C in 100 mM NaCl and 200 mM NaHEPES at pH 7.5. Duplex identity, concentrations of cell and syringe solutions and temperatures at which the data was collected is listed on each figure. Top: Raw curve of energy versus time. Bottom: Fit of integrated peak areas yielding the thermodynamic parameters presented in Table 1 in the main text.

2) Two-Strand Melting Curves and Analysis

**
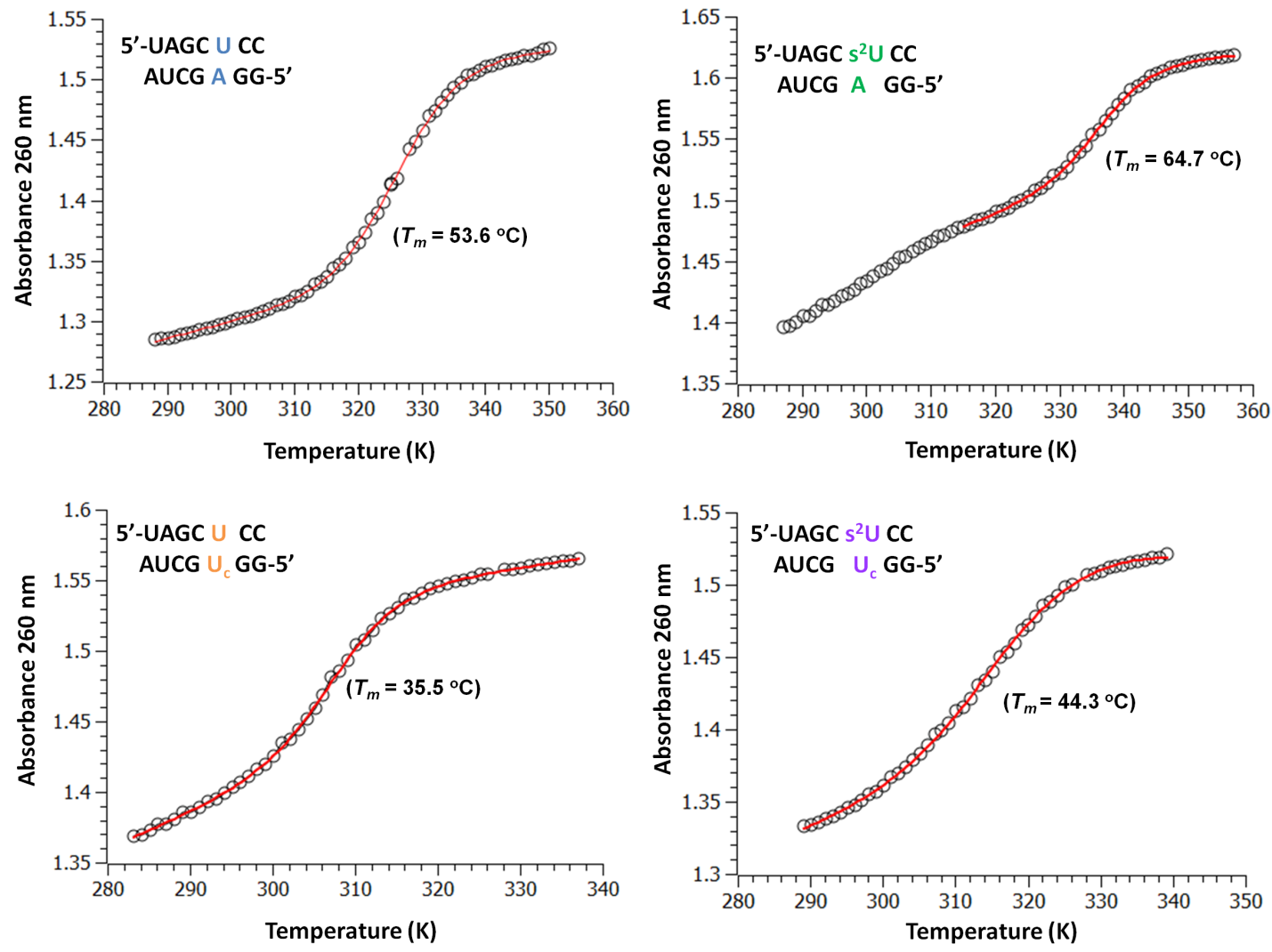
**

**Figure S2.** Melting curves (black circles) and double-baseline, two-state model fits (red lines) collected during the thermal denaturation of 200 μM dsRNA dissolved in buffer containing 100 mM NaCl and 200 mM NaHEPES, pH 7.5. Duplex identities and melting temperatures are listed on the figures. The melting curve of **s^2^U:A** (upper right) exhibits a premelting transition at all oligonucleotide concentrations tested.


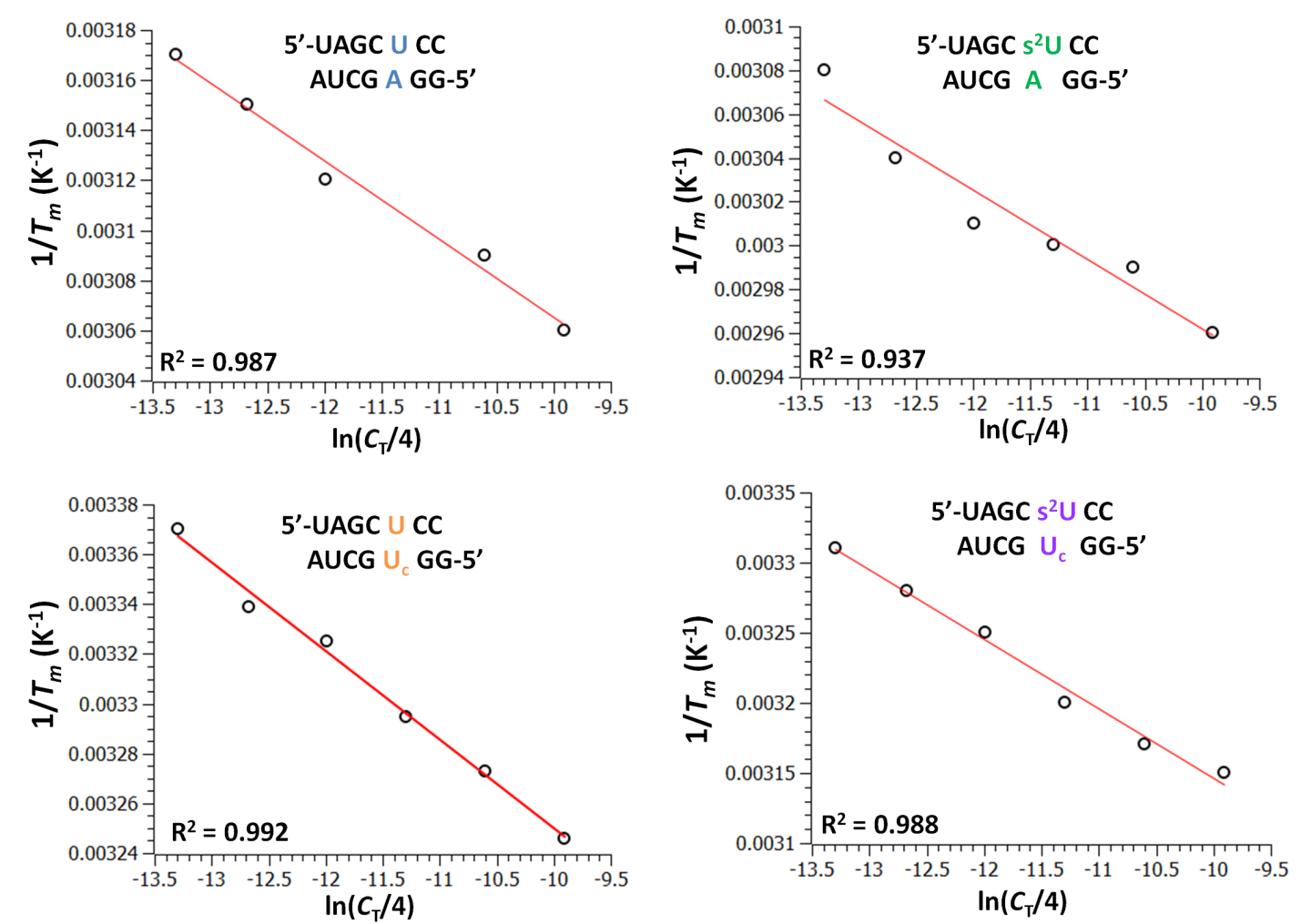


**Figure S3.** Linear least-squared fits (red lines) of van ′t Hoff plots of inverse melting temperatures (*T*_m_^-1^) collected from optical melts at different total oligonucleotide concentrations (*C*_T_) of dsRNA. Duplex identities and R^2^ values are listed on the figures.

3) Single-Strand Melting Curves and Analysis

In order to test the hypothesis that the differences in the observed Δ*H*_ITC_ for **s^2^U:A** and **U:A** in comparison to **s^2^U:U_c_** and **U:U_c_**, respectively, arise from significant amounts of mismatched dimers of the component strands at the beginning of the titrations, as well as to gain insight into the extent of single-strand conformational ordering, we performed thermal denaturation experiments on all four of the single strand components employed in this study using standard UV absorbance methods. The variable concentration melting curves of solutions containing only either the U, s^2^U or A strands are shown in Figure S4. At 200 µM, the U, s^2^U and A strands all show an increase in A_260_ as the temperature is increased, and the shapes of the curves resemble those of typical melting profiles, with the exception that the low temperature initial base-line is not observable. This observation implies that the melting temperature, be it an intramolecular single-strand unstacking process or intermolecular strand-dimerization, is relatively low and hence the sum of the energy of the interactions involved is weak. The lack of an initial low temperature baseline means we are not able to reliably quantify the melting temperatures of any of the solutions containing these three strands. Nevertheless, qualitative information about whether the observed melting process is intra- or intermolecular in nature can still be obtained by carrying out a series of variable concentration melting experiments, as show in Figure S4. For a totally intramolecular process, such as single-strand unstacking, the shape of the melting curve should not depend on concentration other than by a scalar factor. An overlay of the melting curves normalized in intensity by concentration should, in theory, overlap completely, while the same overlay of melting curves for an intermolecular process such as strand dimerization should not. To a large extent, the overlayed normalized curves for both U and s^2^U show a large degree of overlap indicating the process giving rise to the changes in absorption at 260 nm is most likely intramolecular. The small amount of deviation from overlap that is observed may indicate that a small amount of intermolecular interactions are taking place, interactions of which could be having a small effect on the observed Δ*H*_ITC_ values. The majority of the change in A_260_ observed is, however, likely a result of intramolecular single-strand stacking/unstacking dynamics.

In the case of the A strand, however, the normalized overlay of melting curves reveal a more significant degree of incomplete overlap. In particular, there is a concentration-dependent shift of the A_260_ value at 0.75 from higher temperature to lower temperature as the concentration of A is decreased. This shift points to an intermolecular interaction, likely the formation of a weakly bound homodimer with two Watson-Crick GC and two GU wobble pairs. The presence of this dimer in solution during the ITC experiments will cause a decrease in the value of the observed Δ*H*_ITC_ values, which is likely why the observed Δ*H*_ITC_ values for **s^2^U:A** and **U:A** are lower than the corrected values of Δ*H*_ITC_ for **s^2^U:U_c_** and **U:U_c_** (see below). Because we are not able to reliably measure the values of *T*_m_ for the A strand, however, we are unable to provide a quantitative correction for these values for **s^2^U:A** and **U:A**.

In the case of the U_c_ strand, evidence of a mismatched dimerized strand was observed by variable concentration UV melting profiles. At all concentrations, well-defined initial baselines were observed allowing for a reliable measurement of the melting temperature. The melting temperatures decrease as the concentration of U_c_ decreases. We propose that this strand is capable of forming a relatively strong homodimer with two GC Watson-Crick pairs and four GU wobble pairs. A van ’t Hoff plot reveals that the *T*_m_^‒1^ values vary linearly with ln(*C*_T_), and the slope and intercept indicate a Δ*H* and Δ*S* of ‒67.6 kcal mol^‒1^ and 203 cal mol^‒1^ K^‒1^, respectively. The large stability of this dimer must be taken into consideration when coming up with a mechanistic model of duplex formation as monitored by ITC (see below).

Significant differences in the hyperchromicity between s^2^U and U are apparent. If these differences are mostly the result of intramolecular single-strand stacking interactions, then this observation suggests that the conformational dynamics of the s^2^U strand are distinctly different from those of the U strand. Melts performed on hydrolyzed ssRNA under identical conditions displayed no hyperchromicity (Fig. S6A), which is consistent with the hypothesis that the changes in A_260_ are mostly a result of single-strand dynamics.


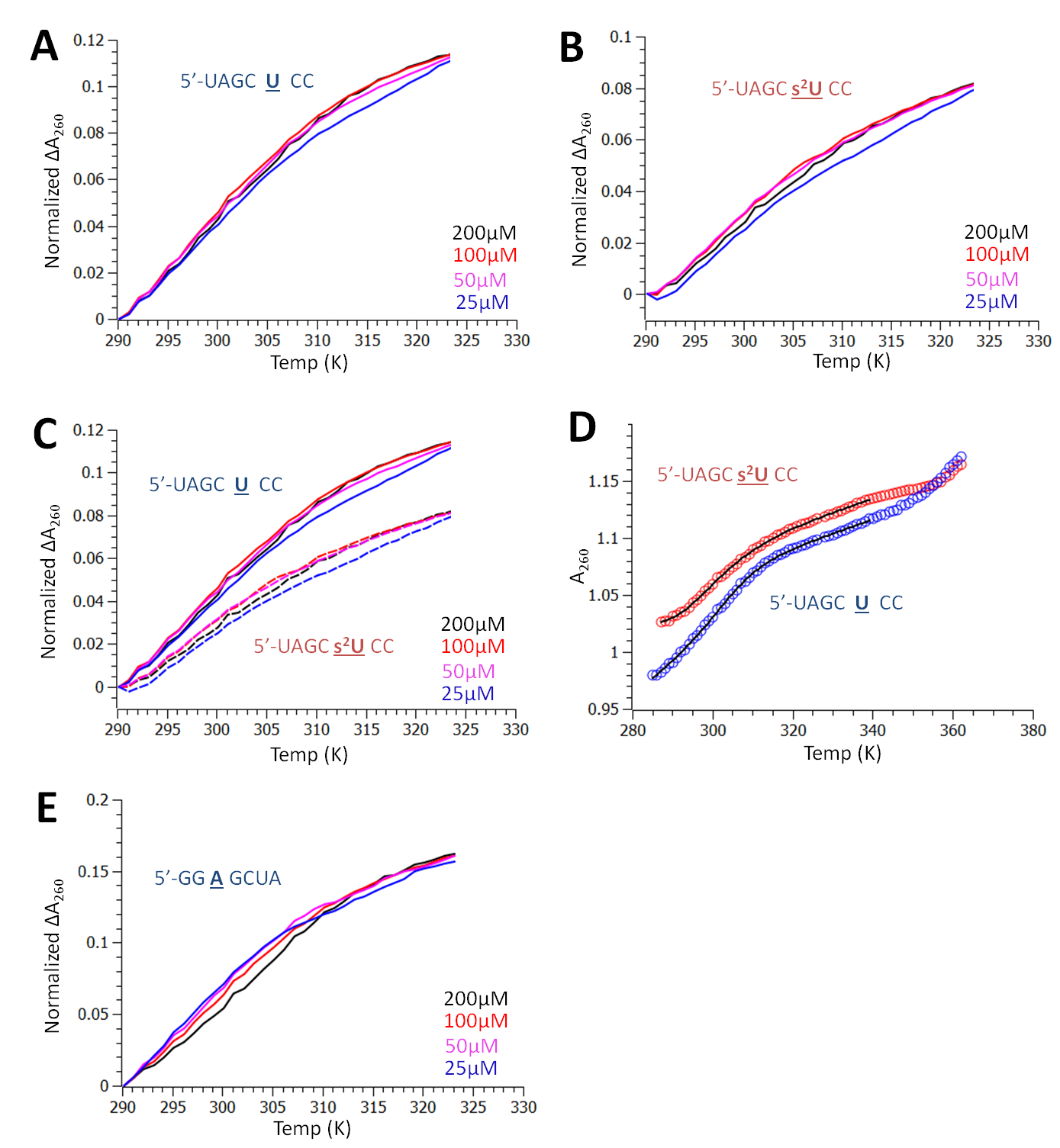


**Figure S4.** Concentration dependant thermal denaturation of ssRNAs. Melts were performed on RNA at 25, 50, 100, and 200 µM in buffer containing 100 mM NaCl and 200 mM NaHEPES at pH 7.5; (**A**) Melting curves collected during the thermal denaturation of U-containing ssRNA; (**B**) Melting curves collected during the thermal denaturation of s^2^U-containing ssRNA; (**C**) Overlapped melts of U-containing ssRNA (solid lines) and s^2^U-containing ssRNA (dashed lines) normalized to the initial absorbance at 260 nm; (**D**) Metls of U-containing ssRNA (blue circles) and s^2^U-containing ssRNA (red circles) and the corresponding double-baseline, two-state model fits (black lines) at 200 µM in buffer containing 100 mM NaCl and 200 mM NaHEPES at pH 7.5. The initial absorbance differences are due to differences in the extinction coefficients between ssRNAs; (**E**) Overlapped melts of A-containing ssRNA (solid lines) normalized to the initial absorbance at 260 nm.


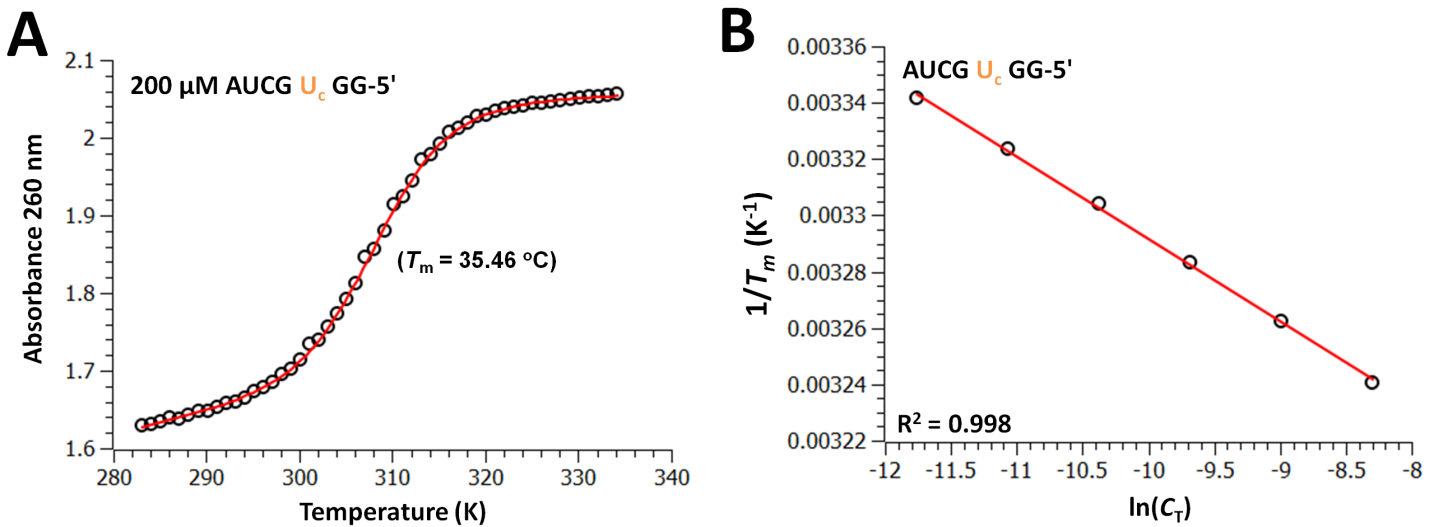
**Figure S5.** UV thermal melting experiments of single-stranded 5′-GGUGCUA. (**A**) Melting curves (black circles) and double-baseline, two-state model fits (red lines) collected during the thermal denaturation of 250 μM of single stranded RNA, 5′-GGUGCUA; (**B**) Linear least-squared fits (red lines) of van ′t Hoff plots of inverse melting temperatures (*T*_m_^-1^) collected from optical melts at different total oligonucleotide concentrations (*C*_T_) of single stranded RNA, 5′-GGUGCUA. The duplex identity and R^2^ value is listed on the figure;

**
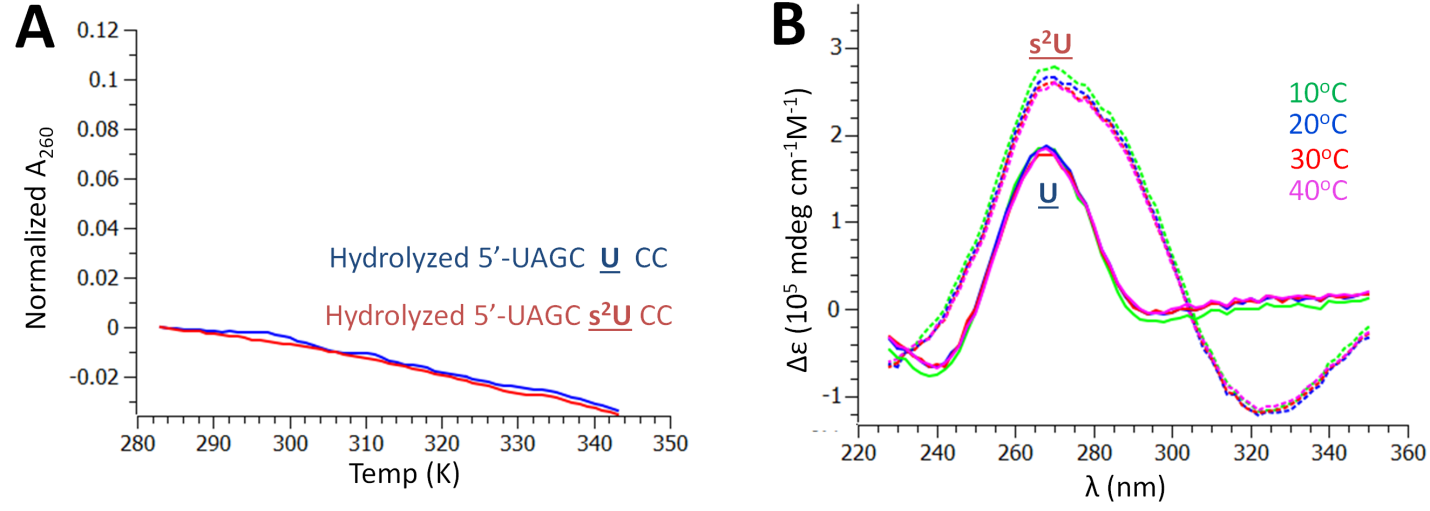
** **Figure S6.** Further investigations into ssRNA secondary structure. (**A**) Overlapped melts of hydrolyzed U-containing ssRNA (red) and hydrolyzed s^2^U-containing ssRNA (blue) at total nucleotide concentrations of 50 µM in buffer containing 100 mM NaCl and 200 mM maHEPES at pH 7.5. Melts were normalized to the initial absorbance at 260 nm. (**B**) Circular Dichroism spectra of ssRNAs at 10–40 °C.

4) pH Titrations

**
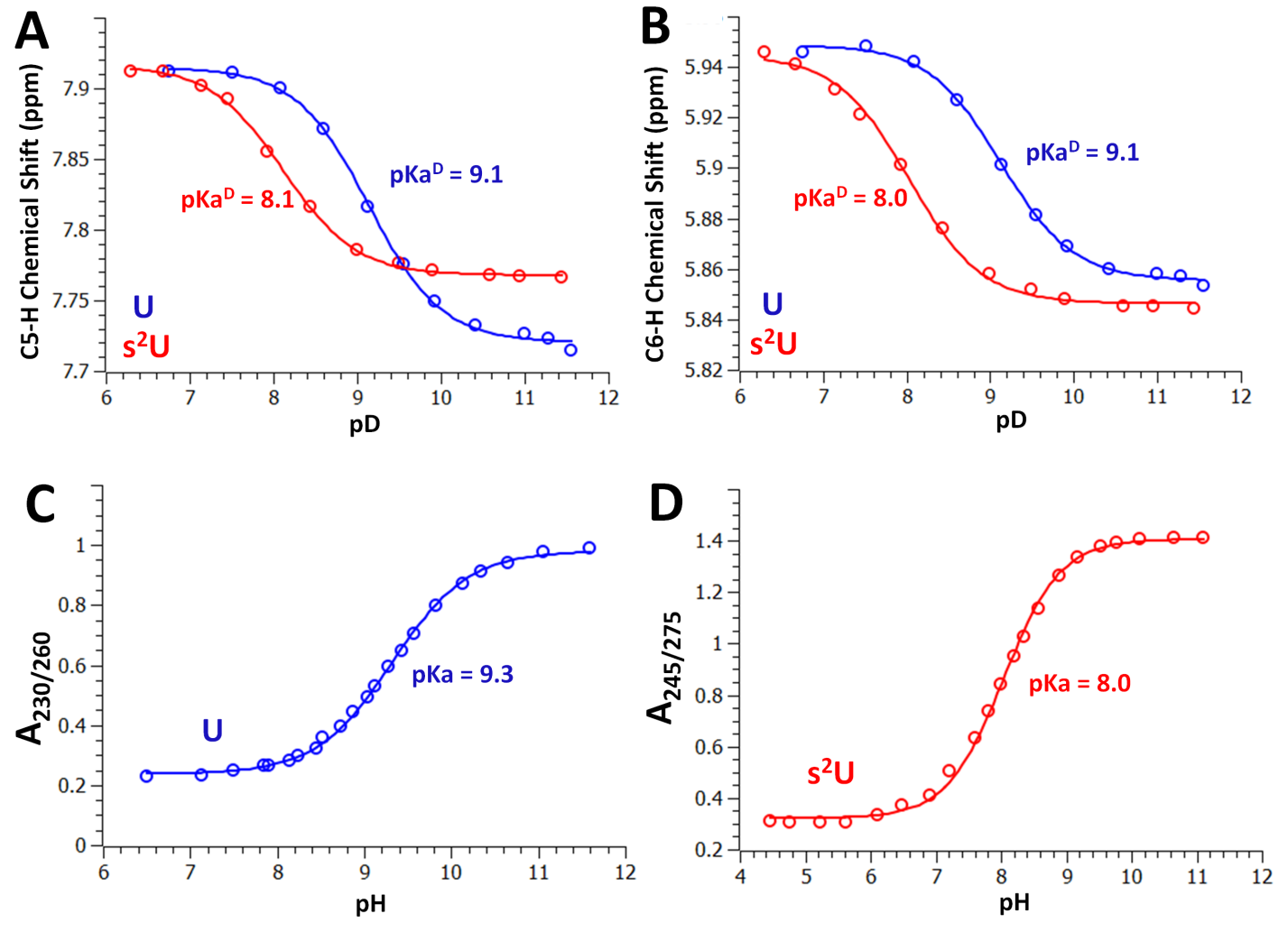
Figure S7.** Determination of pKa for U and s2U nucleosides. Blue markers and lines are for U and red markers and lines are for s^2^U. Lines are sigmoid fits from which values of pKa was calculated from the inflexion point. The R^2^ values of all fits are in excess of 0.99. All titrations were performed at 298 K. (**A**) Normalized NMR chemical shift of the C5 proton as a function of pD. Solutions for NMR contained 10 mM nucleoside dissolved in 500 mM NaCl. Values of pD were measured in D_2_O and have been corrected by adding 0.45 as described by Mikkelsen and Nielsen ([1](#_ENREF_1)); (**B**) Normalized NMR chemical shift of the C6 proton as a function of pD; (**C**) Ratio of absorbance at 230/260 nm for U as a function of pH. Solutions for UV absorbance contained 50 μM nucleoside dissolved in 500 mM NaCl; (**D**) Ratio of absorbance at 245/275 nm for s^2^U as a function of pH.

5) Derivation of the ITC Model

Thermodynamic parameters for **s^2^U:U_c_** and **U:U_c_** duplexes were established by fitting the data to a model designed to compensate for the formation of 3´-aucg**U**gg-5´ (denoted as **U_c_**:**U_c_** below) homo-dimers.

This model begins with an equilibrium statement describing the formation of **U_c_:U_c_** homodimers and the formation of fully complimentary duplexes of **U_c_** and its ssRNA compliment (denoted as **U** below). The fully complimentary duplex is denoted as **U:U_c_**:

(eq1)

Where *L* and *K* are the equilibrium constants governing the formation of the respective duplexes.

The mole fraction of the **U:U_c_** duplex present in solution can then be expressed in terms of concentrations of **U:U_c_**, **U_c_:U_c_** and free **U_c_** as:

(eq2)

Substituting the concentrations in terms of equilibrium constants leads to:

(eq3)

The right hand side of the equation can then be set to a standard two-state binding isotherm with an equilibrium constant, *K*_OBS_:

(eq4)

Solving for *K*_OBS_ gives:

(eq5)

It should be noted that [**U_c_**] is not constant and changes during the titration. However, we can approximate [**U_c_**] to be what it is at the beginning of the titration by solving for **U_c_** when **U** is equal to zero. This approximation has been shown to be valid under similar contexts (2).

(eq6)

By carrying out the ITC titration with **U_c_** in the cell and **U** in the syringe, the observed binding constant *K*_obs_ can be corrected.

The model also needs to correct for the observed change in enthalpy Δ*H*_OBS_. The observed change in enthalpy is equal to the change in mole fractions (Δ*f*s) with respect to the beginning and end of the titration of all species in solution multiplied by their respective *ΔH* terms. Since the initial solution in the cell contains a certain fraction of dimerized **U_c_**, this is accounted for in the following expression:

(eq7)

in which the change in mole fraction is evaluated from zero concentration of **U** to the theoretical limit where [U] is taken to infinity.

(eq8)

The change in mole fraction of **U:U**_c_ is equal to one, while the change in mole fraction of **U_c_:U_c_** depends on the initial concentration at [U] = 0. Writing the mole fraction of **U_c_:U_c_** in terms of *L* and [U_c_] leads to the final expression for Δ*H*_OBS_:

(eq9)

where [U_c_] is its initial concentration in the cell given by equation 6. From the independent measurements of both *L* and Δ*H*_UC:UC_ obtained from UV melting data, Δ*H*_U:UC_ can be calculated.

**Table S1**: The thermodynamic parameters of RNA duplex formation by isothermal titration calorimetry and thermal denaturation.

| duplex | base pair | Δ*H*_ITC_^a^  (kcal mol^-1^) | Δ*ST*_ITC_^b^  (kcal mol^-1^) | Δ*G*_ITC_^c^  (kcal mol^-1^) | ITC  Molar Ratio | Δ*H*_vH_^d^  (kcal mol^-1^) | Δ*TS*_vH_^d^  (kcal mol^-1^) | Δ*G*_vH_^d^  (kcal mol^-1^) |
| --- | --- | --- | --- | --- | --- | --- | --- | --- |
| 5´-uagc**U**cc-3´  3´-aucg**A**gg-5´ | U:A | –47.7(2) | –37.7(3) | –10.0(1) | 0.92(2) | –62.7  (–62.03) | –51.4  (–50.0) | –11.3  (–12.0) |
| 5´-uagc**s^2^U**cc-3´  3´-aucg**A**gg-5´ | s^2^U:A | –45.5(2) | –35.0(2) | –10.5(1) | 0.94(5) | –58.5 | –45.6 | –12.8 |
| 5´-uagc**U**cc-3´  3´-aucg**U**gg-5’ | U:U | –64.3(1)^e^ | –56.1(1)^e^ | –8.18(1)^e^ | 0.90(4) | –54.3  (–51.7) | –46.3  (–44.0) | –7.6  (–7.7) |
| 5´-uagc**s^2^U**cc-3´  3´-aucg**U**gg-5´ | s^2^U:U | –55.0(1)^e^ | –46.0(1)^e^ | –9.05(1)^e^ | 1.10(4) | –40.4 | –32.0 | –8.4 |
| ^a^ **Δ*H*_ITC_** values were evaluated directly from ITC titration data using an independent fit in NanoAnalyze software from TA instruments. ^b^ **Δ*ST*_ITC_** was calculated according to *RT* ln(*K*_d_) = Δ*H* –Δ*ST* where *R* is the gas constant and *T* is temperature. ^c^ **Δ*G*_ITC_** was calculated using values of *K*_d_ from the ITC data according to Δ*G*_ITC_  = *RT* ln(*K*_d_). All ITC titrations were performed in triplicate at 25^°^C. ^d^ **Δ*H*_vH_**, **Δ*TS*_vH_** were derived from linear fits of van ’t Hoff plots of 1/*T*_m_ versus ln(C_T_/4) where C_T_ is the total oligonucle otide concentration and **Δ*G*_vH_** was calculated according to Δ*G*_vH_ = Δ*H*_vH_ –Δ*TS*_vH_. ^e^Values reported have been corrected according to the mechanism derived above. | | | | | | | | |

6) Crystal Structure Analysis

**
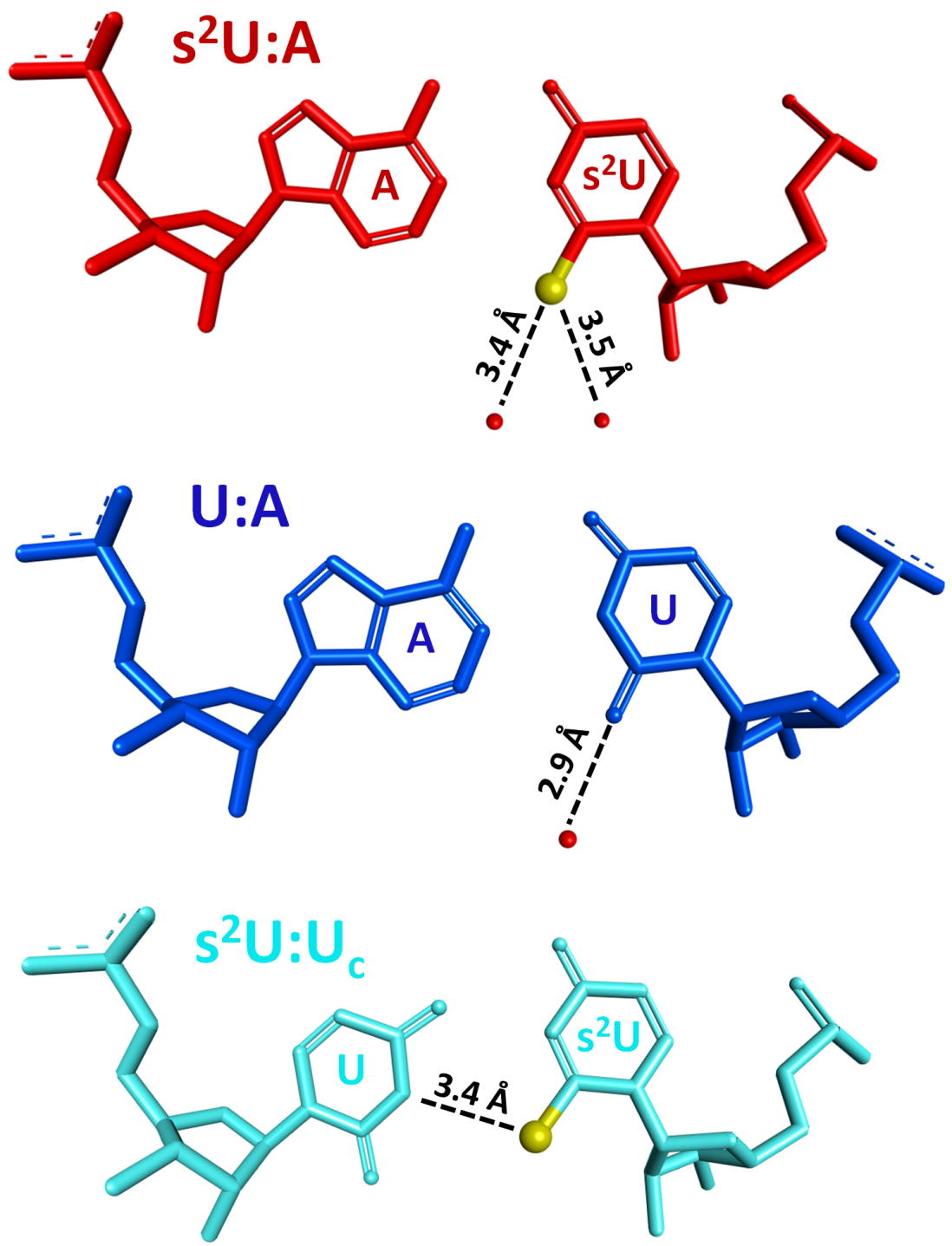
**

**Figure S8.** Measured distances between the S2 and O2 atoms and water O atoms in the RNA duplexes of **s^2^U:A** (red, PDB ID: 4U34), **U:A** (blue, PDB ID: 4U37), and **s^2^U:U_c_** (light blue, PDB ID: 4U35). Sulfur atoms are yellow spheres and water O atoms are red spheres. All water O atoms within 4 Å of the S2 and O2 atoms are displayed. Distances are measured from the center of the water oxygen atoms and the S2 or O2 atoms.

7) Calculations

**APBS Input file (.in):**

# READ IN MOLECULES

read

mol pqr moleculename.pqr

end

elec name solv # Electrostatics calculation on the solvated state

mg-manual # Specify the mode for APBS to run

dime 97 97 97 # The grid dimensions

nlev 4 # Multigrid level parameter

grid 0.33 0.33 0.33 # Grid spacing

gcent mol 1 # Center the grid on molecule 1

mol 1 # Perform the calculation on molecule 1

lpbe # Solve the linearized Poisson-Boltzmann equation

bcfl mdh # was mdh Use all multipole moments when calculating the potential

ion #Na ions at a concentration of 100 mM

charge 1

conc 0.100

radius 2.27

ion #Cl ions at a concentration of 100 mM

charge -1

conc 0.100

radius 1.75

pdie 4.0 # Solute dielectric

sdie 78.54 # Solvent dielectric

chgm spl2 # Spline-based discretization of the delta functions

srfm mol # Molecular surface definition

srad 1.4 # Solvent probe radius (for molecular surface)

swin 0.3 # Solvent surface spline window (not used here)

sdens 10.0 # Sphere density for accessibility object

temp 298.15 # Temperature

calcenergy total # Calculate energies

calcforce no # Do not calculate forces

end

elec name ref # Calculate potential for reference (vacuum) state

mg-manual

dime 97 97 97

nlev 4

grid 0.33 0.33 0.33

gcent mol 1

mol 1

lpbe

bcfl mdh

ion

charge 1

conc 0.100

radius 2.0

ion

charge -1

conc 0.100

radius 2.0

pdie 4.0

sdie 1.0

chgm spl2

srfm mol

srad 1.4

swin 0.3

sdens 10.0

temp 298.15

calcenergy total

calcforce no

end

# Calculate solvation energy

print energy solv - ref end

quit

References:

1. Mikkelsen, K. and Nielsen, S.O. (1960) Acidity measurements with the glass electrode in H2O-D2o mixtures. *J. Phys. Chem.*, **64**, 632-637.

2. Sigurskjold, B.W. (2000) Exact analysis of competition ligand binding by displacement isothermal titration calorimetry. *Anal. Biochem.*, **277**, 260-266.
